# Histone H4 lysine 20 mono-methylation directly facilitates chromatin openness and promotes transcription of housekeeping genes

**DOI:** 10.1038/s41467-021-25051-2

**Published:** 2021-08-20

**Authors:** Muhammad Shoaib, Qinming Chen, Xiangyan Shi, Nidhi Nair, Chinmayi Prasanna, Renliang Yang, David Walter, Klaus S. Frederiksen, Hjorleifur Einarsson, J. Peter Svensson, Chuan Fa Liu, Karl Ekwall, Mads Lerdrup, Lars Nordenskiöld, Claus S. Sørensen

**Affiliations:** 1 Biotech Research and Innovation Centre, Faculty of Health and Medical Sciences, University of Copenhagen, Copenhagen, Denmark; 2 School of Biological Sciences, Nanyang Technological University, Singapore, Singapore; 3 School of Physical and Mathematical Sciences, Nanyang Technological University, Singapore, Singapore; 4 Wilmar International Limited, Jurong Island, Singapore; 5 Global Drug Discovery, Novo Nordisk A/S, Måløv, Denmark; 6 Department of Biosciences and Nutrition, Karolinska Institute, Huddinge, Sweden; 7 Center for Chromosome Stability, Department of Cellular and Molecular Medicine, Faculty of Health and Medical Sciences, University of Copenhagen, Copenhagen, Denmark; 8 Present Address: Department of Biology, SBA School of Science and Engineering, Lahore University of Management Sciences, Lahore, Pakistan

**Keywords:** Gene expression, Chromatin structure, Histone post-translational modifications

## Abstract

Histone lysine methylations have primarily been linked to selective recruitment of reader or effector proteins that subsequently modify chromatin regions and mediate genome functions. Here, we describe a divergent role for histone H4 lysine 20 mono-methylation (H4K20me1) and demonstrate that it directly facilitates chromatin openness and accessibility by disrupting chromatin folding. Thus, accumulation of H4K20me1 demarcates highly accessible chromatin at genes, and this is maintained throughout the cell cycle. In vitro, H4K20me1-containing nucleosomal arrays with nucleosome repeat lengths (NRL) of 187 and 197 are less compact than unmethylated (H4K20me0) or trimethylated (H4K20me3) arrays. Concordantly, and in contrast to trimethylated and unmethylated tails, solid-state NMR data shows that H4K20 mono-methylation changes the H4 conformational state and leads to more dynamic histone H4-tails. Notably, the increased chromatin accessibility mediated by H4K20me1 facilitates gene expression, particularly of housekeeping genes. Altogether, we show how the methylation state of a single histone H4 residue operates as a focal point in chromatin structure control. While H4K20me1 directly promotes chromatin openness at highly transcribed genes, it also serves as a stepping-stone for H4K20me3-dependent chromatin compaction.

## Abstract (web summary)

The effect of histone H4 lysine 20 methylation (H4K20me) on chromatin accessibility are not well established. Here the authors show how H4K20 methylation regulates chromatin structure and accessibility to ensure precise transcriptional outputs through the cell cycle using genome-wide approaches, in vitro biophysical assays, and NMR.

## Introduction

The landscape of chromatin accessibility is a continuum of different chromatin compaction states ranging from facultative and constitutive heterochromatin to more open, accessible euchromatin. The degree of chromatin compaction at any given genomic loci is determined by the nucleosomal packing as well as chromatin binding proteins. Nucleosomes—the fundamental repeating structural units of chromatin, consist of ~147 bp of DNA wrapped around an 8-mer of core histone proteins H2A, H2B, H3, and H4. Nucleosomal packing is influenced by histone composition and histone post-translational modifications at their N-terminal and C-terminal tails, which then modulates chromatin compaction states^1–5^.

The histone H4 lysine 20 (H4K20) methylation pathway has previously been linked to the maintenance of a compact chromatin state^4,6–8^. This pathway is highly cell cycle regulated as H4K20me1, catalyzed by the essential histone methyltransferase SET8, peaks in G2 and M phase^6,9^. H4K20me1 is then further modified to higher methylation states (H4K20me2/3) by SUV4-20H1/2 enzymes in the G1 and S phase of the cell cycle^9–11^. Remarkably, although H4K20me1 and H4K20me3 marks have both been linked to chromatin compaction and transcriptional repression^7,8,10,12–14^, H4K20me1 is predominantly enriched within actively transcribing gene bodies^15–19^. These regions are characterized by a more open chromatin folding state, hence, the H4K20me1 enrichment at these regions appears contradictory to its previously suggested role in chromatin compaction. Moreover, the potential effect of this histone modification on the biophysical properties and compaction of well-defined in vitro reconstituted chromatin fibers and its causal relationship to chromatin accessibility is currently not known. The histone H4-tail basic stretch of amino acids (AA) K16–K23 was previously shown to mediate chromatin folding by interacting with an acidic patch of AAs of the neighboring nucleosome, which leads to nucleosome stacking in folded chromatin fibers^20–22^. Also, acetylation of H4K16 has been shown to abolish the maximal folding of arrays thus creating a mechanism for global decondensation of chromatin regions^22–24^. However, whether the H4K20me1 mark is associated with chromatin fiber unfolding and decompaction is, to the best of our knowledge, unknown.

Histone lysine methylations are best known for providing dynamic binding platforms for a large number of effector proteins that bind through specific domains^25^. These domains recognize the methylated lysine and the adjacent amino acid stretch with high affinity and in turn regulate chromatin functions^2,25^. Consistent with these obervations, it is generally believed that methylated lysines in histone proteins do not directly alter the chromatin structure as, unlike acetylated lysine, there is no net change in charge distribution between methylated and unmethylated lysine^2^. However, it is currently not well understood how a methylated lysine impacts the conformational dynamics of histone tail and globular core domain.

Therefore, to decipher the contribution of H4K20me1 and H4K20me3 states in regulating in vivo chromatin compaction, here we first investigate the link between accumulation of H4K20me1 and chromatin accessibility and its subsequent impact on facilitation of gene expression. Secondly, we characterize the biophysical effect of this mark on the compaction of well-defined in vitro reconstituted chromatin fibers. Finally, we address the role of H4K20me1 in the conformational dynamics of histone H4 tail and globular core domain.

## Results and discussion

### Histone H4 lysine 20 mono-methylation promotes chromatin accessibility at gene bodies

**Fig. 1:**
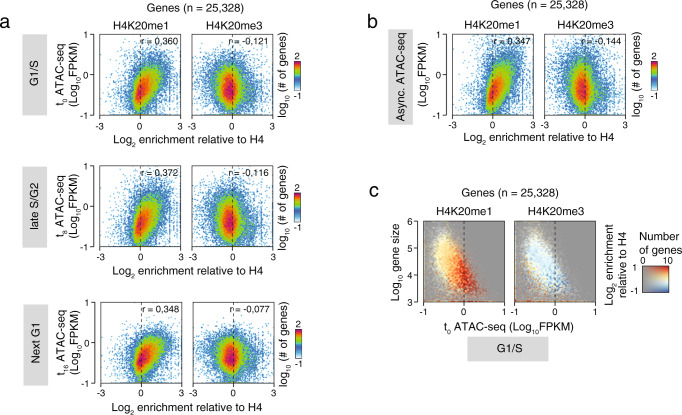
Chromatin accessibility at genes bodies correlates with histone H4 lysine 20 mono-methylation throughout the cell cycle. **a** 2D-histograms showing the relationship between enrichment of selected H4K20 methylation states (*X*-axis) and chromatin accessibility probed using ATAC-seq (*Y*-axis) at human genes. *X*-axis shows log2 normalized H4K20me1 (left side) or H4K20me3 (right side) ChIP-seq levels relative to the levels of H4 control ChIP-seq, while *Y*-axis shows log10-transformed average FPKM-normalized ATAC-seq signal from synchronized U2OS cells at different cell cycle phases, i.e., G1/S (0 h—top, three replicates), S-G2 (8 h—middle, three replicates) and next G1 phase (16 h—bottom, four replicates). The color scales reflect the number of genes having a certain combination of H4K20me enrichment and ATAC-seq. *r*-values show the Pearson’s correlation coefficients for the data in each plot. **b** 2D-histograms showing the relationship between enrichment of selected H4K20 methylation states (*X*-axis) and chromatin accessibility probed using ATAC-seq (*Y*-axis) at human genes as in **a**. *Y*-axis shows log10-transformed average FPKM-normalized ATAC-seq signal from four replicates of U2OS cells grown asynchronously. **c** Colored 2D-histograms showing the three-way relationship between enrichment of selected H4K20 methylation states (color), ATAC-seq signal (*X*-axis), and gene sizes (*Y*-axis) at human genes. Coloring shows log2 normalized H4K20me1 (left side) or H4K20me3 (right side) ChIP-seq levels relative to the levels of H4 control ChIP-seq with the number of genes with a certain combination of ATAC-seq signal and size controlling opacity as indicated in the right side color scale. *X*-axis show log10-transformed average FPKM-normalized ATAC-seq signal from three replicates of synchronized U2OS cells 0 h after release.

We first set out to explore how H4K20me1 and H4K20me3 methylation states vary with chromatin accessibility and compaction of different annotated regions of the genome over the cell cycle. To this end, we synchronized U2OS cells with a double thymidine block and harvested at the G1/S transition (t0), late S/G2 phase (t8), or the next G1 phase (t16) (Supplementary Fig. 1a–f). To measure genome-wide chromatin accessibility at these different phases of cell cycle, we performed ATAC-seq (Assay for Transposase-Accessible Chromatin with high-throughput sequencing)^26^. ATAC-seq relies on hyperactive transposase Tn5, which fragments chromatin genome-wide at accessible locations, followed by next-generation sequencing. Although ATAC-seq mainly captures nucleosome free regions, it has also been shown to enable identification of longer multi-nucleosomal reads and can be used to study a larger range of chromatin accessibililty states at different types of genomic loci^26^. To define H4K20me1 and H4K20me3 enriched loci we performed genome-wide profiling by chromatin immunoprecipitation followed by high-throughput sequencing (ChIP-seq) (Supplementary Fig. 1g). When comparing ATAC-seq and ChIP-seq levels at annotated genes, we found that genes with higher H4K20me1 levels had higher chromatin accessibility than genes with lower H4K20me1 levels (Fig. 1a, b), consistent with enrichment of H4K20me1 at actively transcribing genes^15–19^. In contrast, H4K20me3 enriched loci generally displayed reduced chromatin accessibility (Fig. 1a, b). Notably, these accessibility profiles of H4K20me1 and H4K20me3 loci were preserved throughout the different cell cycle phases and in an asynchronous population of cells (Fig. 1a, b). Supplementary Fig. 1h shows the SET8-kd and H4K20me1 levels in asynchronous U2OS cells. As expected, H4K20me1 was enriched within intragenic and gene overlapping regions compared to intergenic loci, and globally it was negatively correlated to H3K9me3, a histone mark abundantly present in transcriptionally repressed loci. Furthermore, we observed a positive genome-wide correlation between H3K9me3 and H4K20me3 (Supplementary Fig. 1i). In conclusion, these observations indicate a marked association between H4K20me1 and chromatin accessibility in the gene bodies that is preserved throughout the cell cycle.

Additionally, we found a weakly positive correlation between H4K20me1 and accessibility at enhancers and transcription start sites (TSSes)^27–29^ across the different cell cycle phases (Supplementary Fig. 2a, c) and in asynchronously growing cells (Supplementary Fig. 2b, d). The relatively higher correlation at TSSes compared to enhancers indicates that H4K20me1 is more closely linked to genic transcription^15^. Similar to our earlier findings in fission yeast^19^, H4K20me1 was considerably more enriched at shorter genes than the H4K20me3 mark (Supplementary Fig. 3a–c). While both short and accessible genes were more enriched in H4K20me1 compared to larger or less accessible genes, H4K20me1 enrichment was generally more associated to accessibility than gene size (Fig. 1c and Supplementary Fig. 3d–f).

**Fig. 2:**
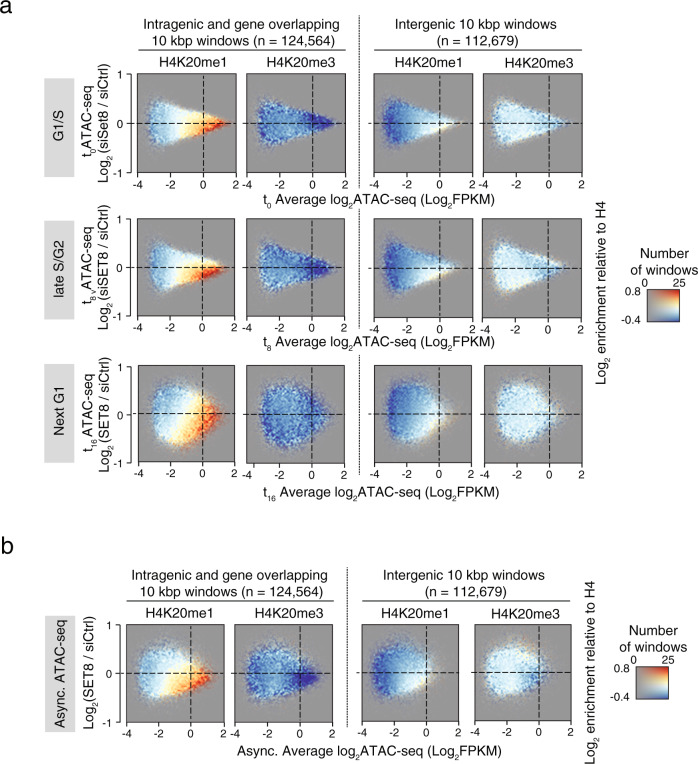
SET8 ensures chromatin accessibility at genes bodies throughout the cell cycle. **a**, **b** Colored 2D-histograms showing the genome-wide three-way relationship between enrichment of H4K20me1 (color) and the level of ATAC-seq signal in SET8-knockdown (kd) over control-kd in U2OS cells. ChIP-seq and ATAC-seq signal was measured in 10 kbp windows and shown separately for windows overlapping fully or partially with annotated gene bodies (leftmost plots) and the windows that did not overlap (rightmost plots). ATAC-seq signal is plotted as MA-plots with average log2 signal from the two conditions on the *X*-axis and the log2 difference between SET8-kd and control-kd on the *Y*-axis. Coloring shows log2 normalized H4K20me1 or H4K20me3 ChIP-seq levels relative to the levels of H4 control ChIP-seq as indicated with the number of windows with a certain combination of ATAC-seq levels (*X*-axis) and change (*Y*-axis) controlling opacity as indicated in the right side color scale. Plots shows FPKM-normalized ATAC-seq signal from cells collected at different cell cycle phases as follows: (**a**) U2OS cells synchronized at G1/S (0 h—top, three replicates), S-G2 (8 h—middle, three replicates) and next G1 phase (16 h—bottom, four replicates), or (**b**) U2OS cells grown asynchronously (four replicates).

We next asked if H4K20me1 is functionally important for maintaining chromatin accessibility. To this end, we examined the accessibility profile of intragenic versus intergenic loci following SET8 depletion, which strongly reduced H4K20me1 levels (Supplementary Fig. 1d, f, h). In addition, we observed general changes in accessibility within gene bodies, where the less accessible 3′ end had consistently reduced accessibility in contrast to the highly accessible 5′ ends (Supplementary Fig. 4). Combined visualization of the changes in accessibility and in H4K20me1 mark revealed that intragenic loci with reduced accessibility after SET8 depletion were generally enriched in H4K20me1, implying that the accessibility of H4K20me1 enriched loci depends on SET8. This was evident at all phases of the cell cycle (Fig. 2a, left panels, bottom-right quadrant). These data point towards a dynamic role of SET8 in promoting chromatin accessibility of intragenic loci throughout the cell cycle by establishing and maintaining H4K20me1 mark. In contrast, H4K20me1 levels were much lower at intergenic loci, and thus SET8 dependency was largely not observed in the intergenic loci (Fig. 2a right panels, bottom-right quadrant). Asynchronously growing cells displayed a similar SET8-dependent chromatin accessibility at H4K20me1 enriched loci (Fig. 2b), supporting the observation that SET8-dependent H4K20me1 primarily mediates chromatin accessibility at intragenic loci. We observed that in synchronized U2OS cells, short-term SET8-kd did not substantially reduce the global H4K20me3 levels (Supplementary Fig. 1d, f). Consistently, the intragenic and intergenic loci with H4K20me3 enrichment were not systematically represented among loci with characteristic changes in chromatin accessibility (Fig. 2a, b).

We next investigated if the highly positive correlation between H4K20me1 and chromatin accessibility at the intragenic, and gene overlapping loci is preserved in other cellular model systems and across mammals. To this end, we performed H4K20me1 ChIP-seq and ATAC-seq in non-transformed mouse embryonic fibroblasts (MEFs) in the presence or absence of SET8 (Supplementary Fig. 5a). Supplementary Fig. 5b illustrates that a considerable fraction of genes in MEF had H4K20me1 enrichment at gene bodies. In line with our findings in U2OS cells, we observed a clear positive correlation between H4K20me1 enrichment and chromatin accessability at intragenic and gene overlapping loci (Supplementary Fig. 5c). Furthermore, chromatin accessibility at these loci was dependent upon SET8 as evidenced by a modest yet significant reduction in accessibility in response to SET8 depletion (Supplementary Fig. 5d, e).

### SET8-H4K20me1 driven chromatin accessibility promotes transcription of housekeeping genes

**Fig. 3:**
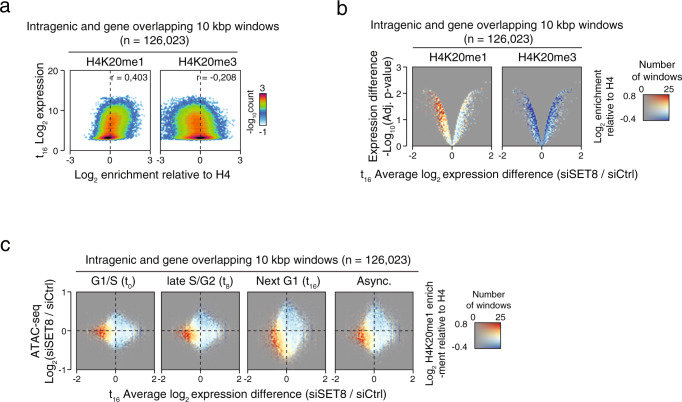
The SET8-H4K20me1 pathway driven chromatin accessibility promotes transcription. **a** 2D-histograms showing the relationship between enrichment of selected H4K20 methylation states (*X*-axes) and expression assayed by microarrays (*Y*-axis) at human genes. *X*-axes shows log_2_ normalized H4K20me1 (left side) or H4K20me3 (right side) ChIP-seq levels relative to the levels of H4 control ChIP-seq, while *Y*-axes shows log_2_-transformed expression levels from synchronized U2OS cells 16 h (G1 phase) after release. The color scales reflect the number of windows having a certain combination of K20me enrichment and expression. *r*-values shows the Pearson’s correlation coefficients for the data in each plot. **b** Colored volcano plots showing the three-way relationship between enrichment of selected H4K20 methylation states (color), gene expression difference (*X*-axis), and significance of the gene expression differences (*Y*-axis). Coloring shows log_2_ normalized H4K20me1 (left side) or H4K20me3 (right side) ChIP-seq levels relative to the levels of H4 control. The opacity in the diagram is controlled by the number of genes having the combination of *X*-axis and *Y*-axis values as indicated in the right-side color scale. *X*-axes show log_2_-transformed difference between SET8-kd over control-kd, and *Y*-axes show –log_10_ adjusted *p*-values from the analysis of the differential expression from two replicates of SET8-kd and three replicates of control-kd in synchronized U2OS cells analysed at 16 h (G1 phase) after release. **c** Colored 2D-histograms showing the genome-wide three-way relationship between enrichment of H4K20me1 (color), expression changes (*X*-axis), and ATAC-seq signal changes in SET8-kd over control-kd in synchronized U2OS. ChIP-seq and ATAC-seq signal was measured in 10 kbp windows that overlapped fully or partially with an annotated gene body. ATAC-seq signal and expression differences are plotted the log_2_ difference between SET8-kd over control-kd. Coloring shows the average log_2_ normalized H4K20me1 ChIP-seq levels relative to the levels of H4 control ChIP-seq as indicated in the right-side color scale. Opacity reflects the number of windows with a certain combination of expression change (*Y*-axis) and ATAC-seq change (*X*-axis) controlling opacity as indicated in the right-side color scale. Plots shows FPKM-normalized ATAC-seq signal from separate rounds of experiments as follows: synchronized U2OS cells at G1/S (0 h—top, three replicates), S-G2 (8 h—middle, three replicates) and next G1 phase (16 h—bottom, four replicates), or U2OS cells grown asynchronously (rightmost, four replicates).

Our data indicated a role for H4K20me1 in maintaining a more accessible chromatin state, which was particularly evident in transcriptionally active regions of the genome. Thus, we investigated the correlation of H4K20me1 and H4K20me3 enrichment to gene expression. We observed a consistent positive correlation of H4K20me1 with the level of gene expression, whereas the inverse correlation was observed for H4K20me3 (Fig. 3a). However, this correlation was much weaker between the intergenic loci and the nearest gene (Supplementary Fig. 6a). Furthermore, we observed an overall weakly positive correlation between gene expression and chromatin accessibility at both intragenic and intergenic loci (Supplementary Fig. 6b). Based on our findings in Fig. 2, we then predicted that gene expression of H4K20me1 enriched genes is SET8 dependent and linked with the ability of the H4K20me1 mark to facilitate an open and accessible chromatin state. Indeed, after SET8-kd, the expression of H4K20me1-positive genes was significantly reduced (Fig. 3b, left panel). Notably, the loci that displayed loss of expression after SET8-kd and showed the most pronounced loss in accessibility had the highest average H4K20me1 enrichment (Fig. 3c, bottom-left quadrant). As expected, there was no observable relationship between H4K20me1 enrichment, chromatin accessibility changes at intergenic loci, and the expression changes of the nearest gene (Supplementary Fig. 6c, left panel and Supplementary Fig. 6d). In contrast to H4K20me1, H4K20me3 enriched loci did not show this relationship between gene expression and chromatin accessibility (Fig. 3b, right panel and Supplementary Fig. 6c, right panel). From these findings, we conclude that SET8-dependent H4K20me1 enrichment at active genes promotes an accessible chromatin state, which in turn is permissive for gene expression. Furthermore, H4K20me1-dependent chromatin accessibility at highly active genes was stably maintained throughout the cell cycle as well as in asynchronously growing cells (Fig. 3c).

These data point towards a role of H4K20me1 in creating an open chromatin environment that in turn facilitates transcription. Previous studies have shown a more direct involvement of SET8 and H4K20me1 in promoting transcription by relieving promotor proximal pausing of RNA polymerase subunit 2, particularly at specific subset of genes^17,30^. Our results complement these studies by showing that in addition to previously shown K20me1-reader dependent mechanism, the K20me1 might directly promote an open chromatin configuration. This way, H4K20me1 adds an additional layer of regulation, whereby H4K20me1 induces an accessible chromatin conformation facilitating gene transcription.

**Fig. 4:**
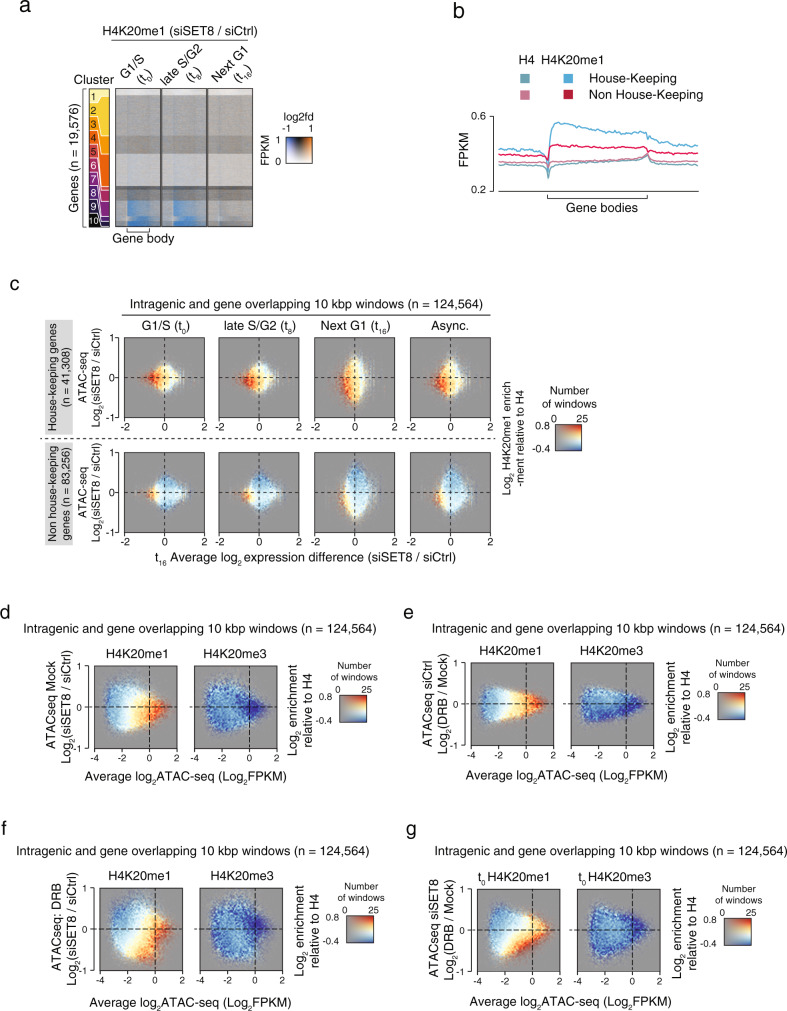
Transcription of housekeeping genes depends on the SET8-H4K20me1 pathway. **a** Heatmaps showing H4K20me1 signal ratios at human genes clustered according to H4K20me1 distribution within the gene bodies in U2OS cells treated with siSET8 and siCtrl. The coloring in the heatmaps is dependent on the total level of signal in both conditions (opacity) and the siSET8/siCtrl ratio, with high and low ratios depicted as orange and blue, respectively. Signal is normalized as FPKMs. Gene bodies are adapted to take up the same visual space regardless of their absolute size in bp, and the horizontal axis in all plots shows the gene bodies as well as upstream and downstream areas corresponding to 50% of gene length. **b** Plots of the average H4K20me1 and H4 FPKM normalized signals at House-keeping genes and genes that are not House-keeping genes. Gene bodies are adapted to take up the same visual space regardless of their absolute size in bp, and the horizontal axis in all plots shows the gene bodies as well as upstream and downstream areas corresponding to 50% of gene length. **c** Colored 2D-histograms showing the genome-wide three-way relationship between enrichment of H4K20me1 (color), expression changes (*X*-axis), and ATAC-seq signal changes in SET8-kd over control-kd in synchronized U2OS as in Fig. 3**c**. The subset of fully or partially gene overlapping windows from Fig. 3**c** were further subdivided into one that specifically overlapped with house-keeping genes (upper panels) or non-house-keeping genes (lower panels). **d**–**g** Colored 2D-histograms showing the genome-wide relationship between enrichment of H4K20methylation enrichment (color) and the changes in ATAC-seq signal as a consequence of SET8-knockdown in untreated (**d**) or DRB-treated (**f**) U2OS cells as well as changes due to DRB-treatment in control (**e**) or SET8 knockdown (**g**) U2OS cells. ChIP-seq and ATAC-seq signal was measured in 10kbp windows and shown for windows overlapping fully or partially with annotated gene bodies. ATAC-seq signal is plotted as MA-plots with average log2 signal from the two conditions on the *X*-axis and the log2 difference between the conditions on the *Y*-axis. Coloring shows log2 normalized H4K20me1 (right plot) or H4K20me3 (left plot) ChIP-seq levels relative to the levels of H4 control ChIP-seq as indicated, with the number of windows with a certain combination of ATAC-seq levels (*X*-axis) and change (*Y*-axis) controlling opacity as indicated in the right side color scale. Plots shows averaged FPKM-normalized ATAC-seq signal from our replicates in each condition of U2OS cells grown asynchronously.

To gain further insights into the transcripts regulated by the SET8-dependent H4K20me1 mark, we investigated if the expression of a specific subset of genes was particularly affected. To this end, we clustered the intragenic H4K20me1 ChIP-seq signal according to the level and distribution along gene bodies in SET8-kd and control-kd conditions. Among the resulting clusters, the most significant transcriptional changes were observed at clusters 7–10 (Fig. 4a and Supplementary Fig. 6e, f), which contained generally small-sized genes (Supplementary Fig. 6g). The gene ontology analysis of these clusters revealed a strong enrichment of pathways involved in basal cellular functions, which mainly contained housekeeping genes (HK) (Supplementary Fig. 7). This subset of genes are constitutively expressed throughout the cell cycle and are critically required for cellular survival^31^. Notably, a set of published HK genes were highly enriched in H4K20me1 mark as compared to non-HK genes (Fig. 4b). We then analyzed the three-way relationship between gene expression levels, chromatin accessibility and H4K20me1 enrichment at the HK genes and compared it to non-HK genes. Interestingly, we found that chromatin accessibility and gene expression of HK genes were strongly dependent on SET8 (Fig. 4c, top panels, bottom left quadrant). Consistently, this relationship remained constant throughout the cell cycle and in asynchronously growing cells. However, the chromatin accessibility and gene expression profile of non-HK genes did not show as strong a dependence on the presence of SET8 and H4K20me1 mark (Fig. 4c, bottom panels). Overall, the analysis of chromatin accessibility of HK versus non-HK genes indicate that SET8-dependent H4K20me1 mark maintains chromatin accessibility of HK genes during different phases of cell cycle and hence, is an important determinant of the constitutive expression of HK genes.

Since, we observed that both accessibility and transcriptional activity of HK-genes are dependent on the presence of H4K20me1 mark, we next explore the possibility that transcriptional activity is the main driving force for maintaining the increased accessibility of H4K20me1 enriched intragenic loci. We, therefore, treated siSET8 and siCtrl U2OS cells with the transcriptional inhibitor 5,6-dichloro-1-β-d-ribofuranosylbenzimidazole (DRB)^32,33^ or mock control for 2 h. DRB acts as a global inhibitor of RNA polymerase II (RNA Pol II)-depedent transcription by preventing activating phosphorylations of the RNA Pol II C-terminal domain^33^. Supplementary Fig. 8a, b shows global transcriptional inhibition after treating U2OS cells ± SET8-kd with DRB inhibitor for 2 h. 5-ethynyl uridine (5-EU) pulse labeling was used to measure the transcriptional activity. For the analysis of chromatin accessibility in the presence of DRB, we generated ATAC-seq libraries from four replicates of each of the four conditions (Mock ± SET8 and DRB ± SET8). Consistent with our previous results, we observed that the accessibility of intragenic loci was dependent upon the presence of H4K20me1 in mock treated cells as loss of SET8 led to loss of accessibility of these loci (Fig. 4d top left and Supplementary fig. 9a). To assess if the transcriptional output of genes impact their accessibility profiles, we next compared ATAC-seq profiles of siCtrl cells treated with either mock or DRB inhibitor. The results indicated that while decreasing the transcriptional output had some impact on the accessibility of genic loci, majority of the loci remained highly accessible (Fig. 4e and Supplementary Fig. 9b) suggesting that active transcription has quite moderate impact on the accessibility of genic regions. We then compared the accessibility profiles of siCtrl vs. siSET8 treated cells in the presence of DRB inhibitor. We reasoned that if transcriptional activity is the main mechanism responsible for maintaining the increased accessibility at H4K20me1 loci, then a combined treatment with DRB and siSET8 in U2OS cells would not be much different from DRB treatment alone. Notably, we observed an aggravated response in DRB-treated siSET8 cells as intragenic loci displayed a further decrease in accessibility in comparison to DRB treatment or siSET8 conditions alone. These data clearly suggest that the accessibility of intragenic loci is dependent upon the SET8-H4K20me1 pathway in a manner, that is not tightly linked to their transcriptional status (Fig. 2f, g and Supplementary Fig. 9c, d).

### Histone H4 lysine 20 mono-methylation results in unfolding of 15-197-601 and 16-187-601 nucleosome arrays

The H4K20me1 mark is not known to be connected to any reader proteins that could immediately explain the increased chromatin accessibility or a decompacted chromatin state. Thus, we hypothesized that H4K20me1 may directly mediate chromatin decompaction, in particular, relative to the repressive H4K20me3 mark. To investigate this, we used in vitro reconstituted nucleosome arrays and performed analytical ultracentrifugation sedimentation velocity (AUC-SV) measurements. The sedimentation coefficient (s_20,w_) measured in this assay is critically dependent on the chromatin fiber size and shape^34,35^. This method therefore enables the detection of differences in the degree of compaction of nucleosome array constructs having varying H4K20 methylation states, and was previously used to establish the abrogating effect of acetylation at H4K16 on nucleosome arrays, thus demonstrating a mechanism for global decondensation of chromatin regions^22–24^. Here, we used the AUC-SV method to elucidate whether the hypothesized effect of H4K20me1 on chromatin folding results in a change of the ability of the nucleosome array to reach the maximally compact state.

We reconstituted chromatin fibers using a DNA array template comprising 15 repeats of 147 bp of the so-called Widom “601” high affinity nucleosome positioning sequence having a nucleosome repeat length (NRL) of 197 bp (15-197-601 DNA) as well as with NRL of 187 bp (16-187-601), which corresponds to linker DNA lengths (nucleosome–nucleosome spacing) of 50 and 40 bp^36^, with recombinant *Homo sapiens* core histones (H2A, H2B, H3 and H4) (Supplementary Fig. 10a). We conducted the installation of methyl-lysine analogs according to established protocols (see Online Methods)^37^ and corroborated the successful installation of the mono-methylation, and tri-methylation constructs by mass-spectrometry (Supplementary Fig. 11a–c). We then applied the AUC-SV method to examine the folding of individual chromatin fibers^23,38^. In addition, constructs with NRL of 202 bp (12-202-601) were prepared and characterized in the same way.

**Fig. 5:**
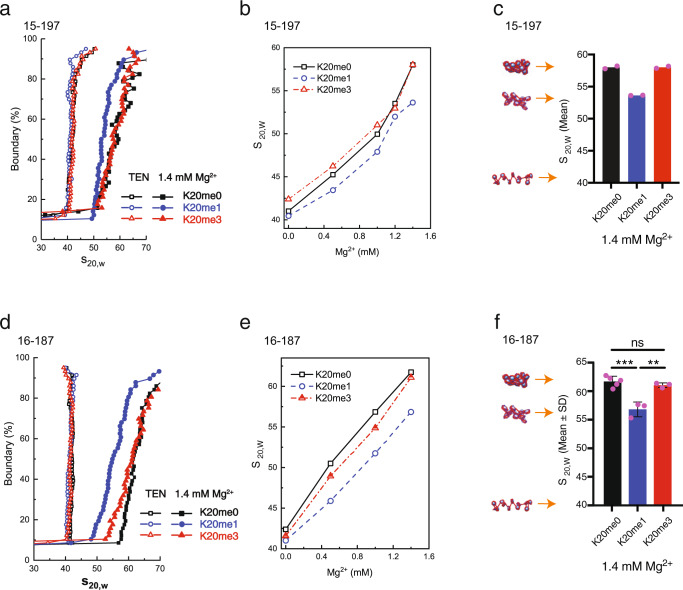
Histone H4 lysine 20 mono-methylation results in unfolding of 15-197-601 and 16-187-601 nucleosome arrays. **a**, **d** van Holde–Weischet curves showing the boundary distribution curves for the 15-197 (**a**) and 16-187 nucleosome arrays (**d**) with the different methylated H4K20 histone constructs in the presence of 1.4 mM Mg^2+^. **b**, **e** AUC-SV titration curves for the 15-197 (**b**) and 16-187 arrays (**e**) in the presence of varying amount of Mg^2+^. Two or three independent sedimentation velocity measurements were performed for each array sample at varying salt concentration. The mean s20,_w_ value was calculated from the s20,_w_ obtained from the average of the values for 20–80% of the boundary curves (corresponding to the curves in **a** and **d**). **c**, **f** Maximal folding of 15-197 (**c**) and 16-187 arrays (**f**) in the presence of 1.4 mM Mg^2+^ for the three H4K20 methylation states. The baseline (at about s20,_w﻿_ = 40S) corresponds to the result in TEN buffer where the arrays adopt a “beads-on-a-string” extended conformation. Trimethylation (K20me3) has no effect on the maximal folding of arrays. The monomethylated array (K20me1) is unable to reach the maximal folding characterized by an *s*-value of 58S for 15-197 array and 61S for 16-187 array. One-way ANOVA analysis for different 16-197 arrays was shown (f). Data are presented as mean values +/− SD. ns not significant, *p* ≥ 0.05; **, 0.001 ≤ *p* < 0.01; ***, 0.0001 ≤ *p* < 0.001. For 16-187 array, *n* = 3 independent experiments for K20me1 and K20me3 arrays; *n* = 5 for K20me0 array; *p* = 0.0007 for me0 vs. me1, and *p* = 0.0056 for me1 vs. me3 (**f**). The representation of the arrays to the left in **c** and **f** schematically illustrates the degree of folding of the arrays. (See the Online Methods section for further details). Source data are provided as a Source Data file.

We analyzed the AUC-SV experiments by the van Holde-Weischet method (Fig. 5a, d and Supplementary Fig. 10b–d). Under buffer conditions and in the absence of added Mg^2+^ both methylated (H4K20me1 and H4K20me3) as well as unmodified (H4K20me0) arrays exhibited the behavior expected for an extended “beads-on-a-string” array conformation with a sedimentation coefficient (s_20,w_) ~ 41-42S for 197 as well as for 187 arrays (Fig. 5b, e). Upon addition of Mg^2+^, the H4K20me0 and H4K20me3 the 197 arrays folded gradually and finally reached the maximal degree of compaction at 1.4 mM Mg^2+^ with a characteristic s_20,w_ ~58S. Remarkably, H4K20me1 exhibited an abrogating effect on chromatin folding; the most compacted state displaying an s_20,w_ value of ~53S at 1.4 mM Mg^2+^ (Fig. 5b, c). This effect of the H4K20me1 mark was somewhat more pronounced for Na^+^-induced chromatin compaction, which displayed a maximal s_20,w_ value of ~50S (Supplementary Fig. 10c). The results for the 187 NRL (Fig. 5e, f) that corresponds to linker DNA length of 40 bp, mirror the findings seen for the 197 construct (with linker DNA length 50 bp), and confirms that this effect is conserved for this shorter NRL that also has a 10n bp nucleosome spacing. These observations suggest that the mono-methylation resulted in a marked disturbance of the H4-tail-mediated nucleosome–nucleosome interaction, which disrupted stacking of folded arrays. The reason that tri-methylation did not show such an effect was likely due to the introduction of the highly hydrophobic group of the K20 side chain. This enables strong hydrophobic interactions with the surface of the neighboring nucleosome, which therefore can maintain the H4 tail-mediated nucleosome–nucleosome stacking characteristic of folded arrays.

We additionally investigated 12-202-601 nucleosome arrays that have a 10n+5 bp rotational setting (linker DNA legth 55 bp). These arrays display considerably less pronounced folding in the presence of Mg^2+^ and there is no effect of the H4K20 methylations (see Supplementary Fig. 12). The result is not unexpected since the changed rotational setting would force the nucleosome–nucleosome stacking orientation to be shifted by 180 degrees. This disrupts the H4 tail interaction with the acidic patch of the neighboring nucleosome leading to less compact arrays, which likely lack the H4 tail mediated interaction that is disrupted by H4K20me1 methylation in the case of 187 and 197 arrays. A study of the Mg^2+^ induced compaction behavior previously showed such a reduced folding for 172 compared to 177 nucleosome arrays^39^. It was also recently shown that 601 chromatin fibers close to 10n+5 bp rotational setting displayed reduced stacking energy and fibers that are more flexible, compared to constructs close to a 10n bp rotational setting^40^.

The observation that the H4K20me3 modification had negligible effect on fiber compaction was different from an earlier report^41^ for 12-207-601 nucleosome arrays, where H4K20me3 resulted in somewhat more compact and oligomerized nucleosome arrays compared to the disruption in folding seen for H4K20me1 in the present work. This difference may be a result of different sample preparation (the previous study used *Xenopus Laevis* histones) or due to the longer nucleosome repeat lengths applied in the experiments, which has previously been shown to result in less compact nucleosome arrays in the presence of Mg^2+^^39,42^. The choice of a NRL of 187 and 197 in the present work was made based on the analysis by Widom of a large number of measurements of NRL in eukaryotic cells, which deduced that values close 187 and 197 are the most abundant^36^. although it should be noted that a variation in observed NRL in the range between 190–200 bp is common in human cells^42^.

Next, we conducted precipitation assays (PA) to examine the efficiency of Mg^2+^ cations to induce self-association of arrays due to fiber–fiber interactions^34,35^. As we established from the AUC-SV measurements, addition of salt to a buffer solution of arrays resulted in a gradual folding of individual arrays until the fibers reached the maximally folded state described above. Further addition of salt to maximally folded arrays resulted in array self-association and aggregation (precipitation) (Supplementary Fig. 13a–c). We observed a modest but significant difference whereby the H4K20me1 arrays required the highest concentrations of Mg^2+^ necessary to result in fiber-fiber interaction and self-association (Supplementary Fig. 13a–c). This result was consistent with the AUC-SV data and suggested that mono-methylation on H4K20 disrupts the attraction between fibers caused by histone tail–DNA interactions^43^. Taken together the AUC-SV and PA data strongly suggest a direct role of H4K20me1 mark in preventing the maximal folding of nucleosome arrays, as well as reducing fiber–fiber interactions thereby creating a more open chromatin state. Importantly, this in vitro analysis supports our cell biological data where the H4K20me1 mark functionally correlated with highly accessible chromatin loci (Fig. 1). It should be noted that recent investigations suggest that chromatin fiber self-association (oligomerization) may be the main contributor to chromatin structure in vivo, which may lack distinctly folded 30 nm fibers^44^. The present data illustrates how the H4K20me1 mark affects both the folding of individual arrays as well as fiber self-association.

### Solid-state NMR reveals change in conformational state of histone H4 tail upon K20 mono-methylation

The AUC-SV results showed that mono-methylation of histone H4K20 results in array unfolding. We therefore reasoned that this could be due to an effect on the interaction of H4 tail with neighboring nucleosomes, which mediates nucleosome–nucleosome stacking and facilitates fiber compaction. Accordingly, we hypothesized that H4K20me1 results in changes in the dynamic conformational state of the histone H4 tail. To investigate this at the molecular level, we performed solid-state NMR (ssNMR) studies of the histone H4 in folded and precipitated 197 nucleosome arrays and compared the dynamic behavior of H4K20me0, H4K20me1 and H4K20me3 arrays. The ssNMR experiments enable AA site-specific information on the dynamic and structural properties of the histone to be assessed and compared for arrays with different H4K20 methylation states.

**Fig. 6:**
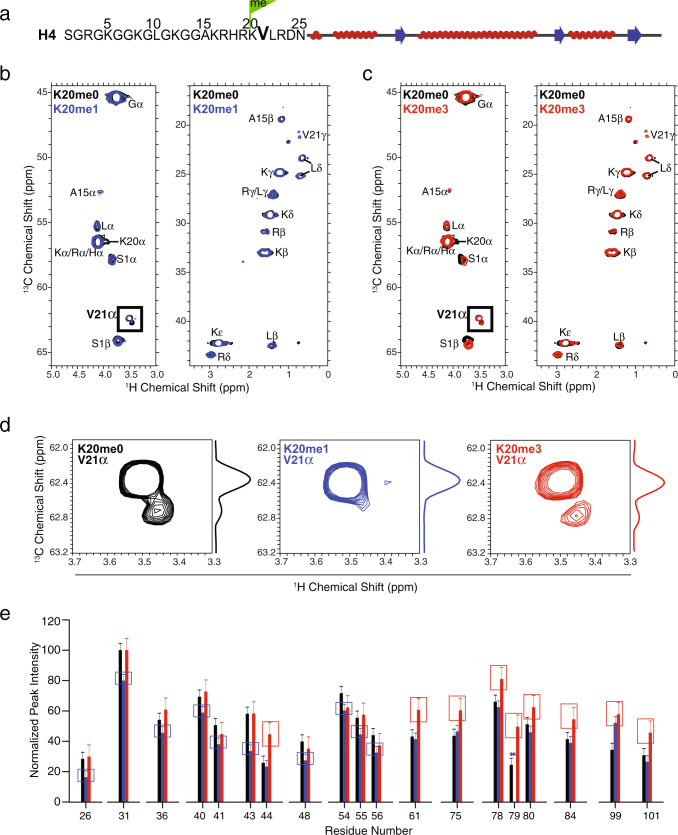
Solid-state NMR reveals change in conformational dynamics of the histone H4 tail upon lysine 20 mono-methylation. **a** Schematic representation of the AA sequence of human histone H4. The green flag highlights the position of AA K20 that is subject to methylation. Overlaid 2D ^1^H-^13^C refocused J-based INEPT spectra of 15-mer nucleosome arrays containing **b** H4K20me0 (black) and H4K20me1 (blue) and **c** H4K20me0 (black) and H4K20me3 (red) with uniformly ^13^C/^15^N labeled H4. The absence of K20 ^1^Hα−^13^Cα correlations in the spectra (**b**, **c**) confirms that the K20me1 and K20me3 modifications of H4 were successful. **d** Zoomed-in plots of AA V21 showing the ^1^Hα−^13^Cα peaks of the three arrays. **e** AA site-specific peak intensities obtained from the two-dimensional (2D) NCA spectra (Supplementary Fig. 14). Peak intensities were normalized to the highest peak intensity of the NCA spectrum, error bars were derived from the RMSD value of the noises in the spectra. The NCA (correlation achieved by magnetization transfer from the N to alpha Cα) and the NCO (correlation achieved by magnetization transfer from the N to carbonyl CO) peak intensities report on the relative AA site-specific dynamic behavior. Here, only H4K20me1 (blue) or H4K20me3 (red) residues having considerable peak intensity differences compared with H4K20me0 (black) nucleosome arrays are displayed. The blue and red boxes highlight the residues exhibiting differences for the H4K20me1 and H4K20me3, respectively, in comparison with the H4K20me0 arrays. The complete peak intensity profiles are shown in Supplementary Fig. 15; the asterisk indicates that the peak intensity is not extracted because of overlapping peaks.

First, in order to probe the conformation and dynamics of the H4 tail in the tightly compacted and precipitated arrays, we collected ^1^H-^13^C/^15^N correlation ssNMR spectra in order to detect those AAs in the H4 tail region, which exhibits significant mobility compared with the rigid core region^45,46^. By using the known averaged chemical shift values of AAs combined with three-dimensional (3D) solution-state NMR experiments of the histone H4 in a Widom “601” nucleosome core particle^47^, we established the tail peak chemical shift assignments. Interestingly, two peaks, 3.50–62.4 ppm and 3.45–62.8 ppm, were observed in the ^1^H−^13^C spectra of the nucleosome arrays containing H4K20me0 or H4K20me3 (Fig. 6a–d). These peaks could be uniquely assigned to the AA Valine 21 (V21) ^1^Hα-^13^Cα because this is the only Valine in the H4 N-terminal tail (Fig. 6a). Furthermore, we unambiguously assigned the H4V21 peak, which made it unique in contrast to the other AA in the tail region that all displayed considerable overlap. Additionally, V21 is located in the middle of the basic AA stretch 16–23 between the more flexible residues towards the N-terminal tail and the significantly more rigid ones closer towards the globular domain, which makes it exclusively positioned to report on the tail conformational dynamics. The two distinct V21 ^1^Hα–^13^Cα peaks in the 15-mer nucleosome arrays containing H4K20me0 or H4K20me3 strongly suggested the presence of two conformations of the tail, referred to as State 1 and State 2, respectively. These two peaks potentially originate from two different tail positions that may correspond to the tails of the two H4 copies in a histone octamer (HO). This is consistent with crystal structure studies of the nucleosome core particle demonstrating that only one of the two H4 tails in the HO is involved in the interaction with the H2A-H2B acidic patch^48^. In contrast, as shown in Fig. 6b, d, the V21 ^1^Hα–^13^Cα peak intensity of State 2 was negligible in the H4K20me1 containing array, indicating that the H4 tails predominantly existed as State 1, suggesting that the interaction between one H4 tail with the H2A-H2B acidic patch is disrupted and, therefore, both tails possess the same conformational state. This observation implies that the partial unfolding of the H4K20me1 array leads to a more open and dynamic chromatin fiber, a result that is corroborated by the more dynamic H4 core region (see below).

Secondly, we acquired dipolar-based ssNMR spectra of compact folded arrays to identify the H4 AA sites within the rigid core region in order to establish any dynamic changes introduced by the different H4K20 methylation states. First, we noted that the overall structures of the H4 globular domains in the three different nucleosomes arrays were identical as evidenced by the 2D spectra (Supplementary Fig. 14a–c). By extracting the peak intensities of the so-called NCA (N to alpha Cα) and NCO (N to carbonyl CO) spectra (Fig. 6e and Supplementary fig. 15a, b), we obtained information about the internal dynamics of the H4 protein backbone at the μs-ms timescale^47,49^. In comparison with the H4K20me0 nucleosome array, the globular domain of the H4K20me1 array showed more uniform peak intensities across the protein and reduced relative intensities for several residues primarily residing in the LN and L1 regions and in the adjacent stretch of the α1 and α2 regions (Fig. 6e and Supplementary Fig. 15a, b). This implied that these core regions of H4K20me1 were more mobile compared to the H4K20me0 arrays, likely related to a higher flexibility of DNA. In contrast, compared with the H4K20me0 arrays, the H4K20me3 construct displayed enhanced relative peak intensities for many residues particularly in L2 and at the end of the C-terminus, which suggested a somewhat more rigid H4 globular domain, possibly correlated with less mobile DNA.

These above results are remarkable as they show that a single H4K20 mono-methylation, which globally causes unfolding of the chromatin fiber, at the molecular level is coupled with both a change in the conformational dynamics of the tail itself as well as it propagates change to the histone H4 globular core dynamics. Taken together, mono-methylation of H4K20 in the nucleosome array uniquely changes the H4 tail conformational population, shifting it from two states to one state as well as resulting in a more flexible histone. Trimethylation of H4K20 does not affect the H4 tail conformation, which remains similar to that of H4K20me0 arrays, albeit resulting in more rigid H4 core region.

**Fig. 7:**
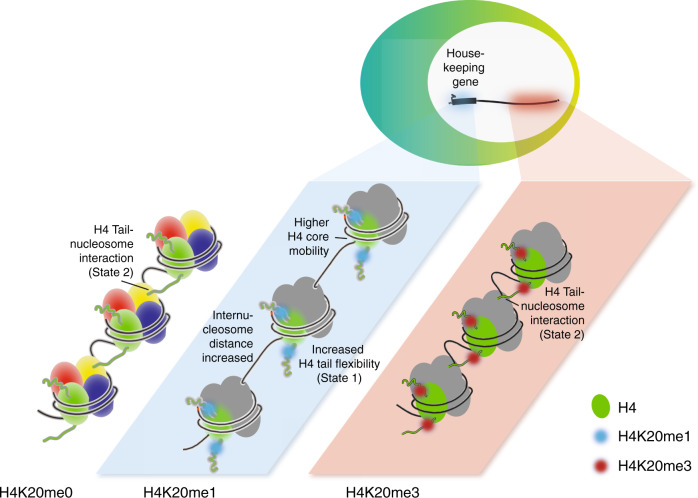
SET8-H4K20me1 pathway promotes housekeeping gene transcription through chromatin structure modulation. Detailed model depicting the chromatin conformation at (**i**) state 1 and (ii) state 2. In state 1, transcription of housekeeping genes is promoted by dynamic H4 tails having H4K20me1 enrichment creating an open chromatin environment. State 2 represents intergenic regions with more static H4 tails having H4K20me3 enrichment. This creates a more compact chromatin which is transcriptionally repressive. In the absence of SET8, expanding H4K20me0 regions promote H4 tails more similar to the H4K20me3 conformation (state 2) resulting in more compact chromatin environment repressive towards transcription.

Based upon the cell biological and structural data presented here, we present a model outlining how the SET8-H4K20me1 pathway impacts the structural and functional characteristics of HK genes (Fig. 7). We propose that HK genes are highly enriched in H4K20me1, which owing to its structurally more dynamic nature dictates a more open and accessible local chromatin environment. This type of chromatin structure in turn facilitates constitutive expression of HK genes. However, in the absence of SET8, HK genes lose H4K20me1 mark leading to a more static, compact, and transcriptionally less permissive chromatin environment. In conclusion, our findings highlight the structural and functional demarcation between the closely linked H4K20me states. We show that the cell cycle regulated H4K20me1 accumulation induces a more accessible, less compact chromatin that facilitates transcription. The mono-methylated state is a stepping stone towards H4K20me3 in a subset of genomic regions, which in turn may induce a more compact chromatin folding state acting as an impediment to transcriptional activities. While histone methylation is a well-known recruitment mechanism for protein binding, our cell biological and biophysical data provide strong evidence that H4K20 mono-methylation can also alter the basic biophysical properties of chromatin and in turn has profound effects on crucial parts of the genome. Our research, therefore, opens up possibilities for further investigations into the role of additional histone methylation marks in directly contributing to chromatin accessibility regulation.

## Methods

### Cell culture and treatments

U2OS cells were obtained from ATCC while Mouse embryonic fibroblasts were a kind gift from Prof. Dr. Gunnar Schotta, Biomedical Center and Center for Integrated Protein Science, Ludwig-Maximilians-University, Munich, Germany. Both cell lines were cultured in Dulbecco’s modified Eagle’s medium containing 10% fetal bovine serum and 1% Penicillin–Streptomycin. MEF cells were in addition supplemented with non-essential amino acids. For synchronization at G1/S, U2OS cells were cultured in the presence of 2 mM thymidine (Sigma) for 20 h, washed two times with phosphate-buffered saline (PBS), and released in fresh medium without thymidine for 10 h. Two millimolar thymidine was then added and cells were cultured for another 17 h. Cells were treated with either scrambled siRNA sequence (siCtrl) or siRNA against *SET8* (siSET8) during the second block 6 h prior to second release (t0—6 h). Cells were then washed two times with PBS and either harvested (t0—G1/S) or released in fresh medium. For analyzing cells in the subsequent S/G2 and next G1 phase, cells were collected after 8 (t8—late S/G2) and 16 h (next G1). For experiments in asynchronously cultured U2OS and MEFs, cells were plated and reverse transfected with either control or SET8 siRNA for 6 h. Fresh media was then added, and cells were incubated for up to 36–48 h (U2OS) or 72 h (MEF) in total from the time of transfection. siRNA transfections were performed with 20 nM siRNA duplexes using Lipofectamine® RNAiMAX (Invitrogen), according to the manufacturer’s instructions. The siRNA sequences used for knockdown are (5′-3′): SET8 (GUACGGAGCGCCAUGAAGU) and SET8 mouse/human (GCUGCAGUCUGAAGAAAGG). U2OS cells were treated either with mock (DMSO only) or with transcriptional inhibitor DRB (5,6-Dichloro-1-beta-Ribo-furanosyl Benzimidazole) at final concentration of 100 µM for 2 h prior to harvesting the cells. For nascent RNA labeling, U2OS cells were pulse labeled with 5-ethynyl uridine (5-EU) (5 mM) for 1 h before fixation.

### Flow cytometry

Cells were fixed in 70% ethanol and DNA was stained using 0.1 mg/ml propidium iodide containing RNase for 30 min at 37 °C. Flow cytometric analysis was performed on FACSCalibur using the CellQuest Pro software (BD). Data were analyzed using the FlowJo software (v10.6.2; TreeStar). At least 10,000 cells were analyzed for each sample. FL2-A vs. FL2-H was used to gate for single cells. A narrow diagonal gate was used to select single cell population. All cells outside of this gate were selected out as they represent either doublets or dead cells. The cell cycle profiles shown in Supplemental Fig. 1c, e represents the cell population from this single cell gate.

### Immunoblotting and antibodies

Cells were lysed on ice in cold EBC-buffer (150 mM NaCl; 50 mM TRIS pH 7.4; 1 mM EDTA; 0.5% NP-40/Igepal) containing protease inhibitors (EDTA free-Protease inhibitor cocktail, 1 mM PMSF, 50 mM NaF) and 1 mM DTT. The lysates were sonicated using Bioruptor Plus sonication device on “High” mode for 10 cycles each of 30 s ON/30 s OFF. Proteins were separated by SDS-PAGE and transferred to a nitrocellulose membrane. Blocking and blotting with primary antibodies were performed in PBS-T supplemented with 5% skimmed milk powder. Proteins were visualized on films using secondary horseradish peroxidase-conjugated antibodies and ECL (GE Healthcare). Films were developed using an X-ray machine (Valsoe; Ferrania Technologies). The primary antibodies used in this study are anti-Vinculin (Sigma; V9131—1/25000), anti-SET8 (Millipore; 06-1304—1/1000), anti-H4K20me1 (Abcam; ab9051—1/5000), anti-H4K20me3 (Diagenode; C15410207—1/1000), anti-H4 pan (Millipore; 04-858—1/5000).

### Immunostaining

Cells grown on 12 mm coverslips were washed twice with PBS, and then fixed with 4% formaldehyde for 10 min at RT. Cover slips were subsequently incubated in blocking solution (1× PBS containing 0.1% Triton X-100 and 3% bovine serum albumin) for 1 h at RT. For EU detection Click-it reaction was performed by incubating the fixed cells in Click-it buffer (100 mM Tris-HCl pH 8, 2 mM CuSO4, 1 ng Alexa Fluor 647 Azide (Life Technologies), and 100 mM ascorbic acid for 30 min at RT. Coverslips were washed three times with 1× PBS and incubated in DAPI (0.5 mg/ml; Sigma-Aldrich) for 10 min at RT. Coverslips were washed three times with 1× PBS and dipped in distilled water, placed on 3 mm paper to dry, and mounted on 5 µl fluorescent mounting media (Dako).

### Quantitative image-based cytometry (QIBC)

Images used for QIBC were obtained automatically with the ScanR acquisition software controlling a motorized Olympus IX-83 wide-field microscope. The system was equipped with filter cubes compatible with DAPI and Cy5 fluorescent dyes, a Spectra X-LIGHT engine Illumination system with six color LEDs and emission filters, and a Hamamatsu Camera Orca Flash 4.0 V2. An Olympus Universal Plan Super Apo 10× Objective was used for all QIBC data. Images were processed using the ScanR image analysis software. TIBCO Spotfire software was used to plot total nuclear pixel intensities for DAPI (Arbitrary units: arb.u.) and mean (total pixel intensities divided by nuclear area) nuclear intensities (arb.u.) for EU intensity in color-coded scatter diagrams in a flow-cytometry-like fashion.

### ChIP-seq

Twenty million cells cultured U2OS cells were fixed with 1% formaldehyde on tissue culture plates at room temperature (RT). Fixation was stopped by addition of glycine to final a concentration of 0.125 M and incubate for 5 min. Plates were rinsed twice with 1× PBS at RT. PBS was completely aspirated from the plate and cells were harvested in SDS Buffer (100 mM NaCl, 50 mM Tris-Cl, pH8.0, 5 mM EDTA, pH 8.0, 0.2% NaN_3_, and 0.5% SDS). Cells were pellet by spinning for 10 min at 500 g. The cell pellet is resuspended in appropriate volume of ice-cold IP Buffer for sonication (IP buffer = 1 volume SDS Buffer: 0.5 volume Triton Dilution Buffer (100 mM Tris-Cl, pH 8.6, 100 mM NaCl, 5 mM EDTA, pH 8.0, 0.2% NaN_3_, 5.0% Triton X-100)). Samples were sonicated to an average length of 300–1000 bp using bath-based Bioruptor sonicator (10–12 cycles—high mode—30 s ON/30 s OFF—4 °C). the sonicated chromatin is centrifuged at 20,000×*g* for 30 min. After verifying the correct fragment size, chromatin was quantified using Bio-Rad Protein Assay Kit II (Cat #5000002). For each individual ChIP, 0.5 mg chromatin was precleared using 40 µl of Protein A Sepharose beads (rProtein A Sepharose® Fast Flow—GE Healthcare). One percentage input was taken out after pre-clearing and the lysates were incubated overnight (ON) with 5 µg of primary antibody in rotation at 4 °C. BSA blocked Protein A Sepharose beads were added after ON incubation with primary antibody and samples were rotated for 3–4 h at 4 °C. Beads were subsequently collected by spinning at 1000 g for 2 min and washed three times with low salt wash buffer (150 mM NaCl, 20 mM Tris-HCl, pH 8.0, 5 mM EDTA, pH 8.0, 5% w/v sucrose, 0.02% NaN_3_, 1% Triton X-100, 0.2% SDS), twice with high salt wash buffer (0.1% (w/v) deoxycholic acid, 1 mM EDTA pH 8.0, 50 mM HEPES, pH 7.5, 500 mM NaCl, 1% (v/v) Triton X-100 and 0.02% NaN_3_), twice with LiCl wash buffer (0.5% (w/v) deoxycholic acid (sodium salt), 1 mM EDTA pH 8.0, 250 mM LiCl, 0.5% (v/v) NP-40, 10 mM Tris-HCl pH 8.0 and 0.02% NaN_3_) and once with TE buffer (10 mM Tris-HCl and 1 mM EDTA pH 8.0). Input samples and beads were then resuspended in decrosslinking buffer (1% SDS, 100 mM NaHCO_3_) and shaken at 65 °C for O/N at 900 rpm in thermoshaker (Eppendorf® Thermomixer Compact—T1317). DNA was then purified using QIAquick PCR purification kit (Cat No./ID: 28106). Chipped DNA was quantified using Qubit DNA HS Kit and 10 ng DNA was taken for prepping libraries for next generation sequencing using iDeal ChIP-seq kit (Cat. # C01010059), and sequencing was done on a Hiseq2000 machine with read length 55 bases.

Native ChIP was performed for histone modifications in MEFs wherein 3 million MEFs were harvested by trypsinization. Following trypsinization, all steps were performed on ice unless stated otherwise. Cell pellet was resuspended in cytosolic lysis buffer (10 mM Tris-HCl, pH 7.5, 10 mM NaCl, 5 mM MgCl_2_, 0.5% NP-40, with added protease inhibitors) and incubated on ice for 8 min. Nuclei pellet was obtained by centrifugation at 1700×*g* for 10 min. The pewllet was washed once in nuclei buffer (60 mM KCl, 15 mM NaCl, 0.34 M sucrose, with added protease inhibitors) and then centrifuged again at 1700×*g* for 3 min. The nuclei pellet was resuspended in nuclei buffer. CaCl_2_ was added to a final concentration of 2 mM to each of the samples. 5U of Micrococcal nuclease (0.2 U/µl; Sigma Aldrich) was added to pre-chilled Eppendorf tubes, followed by samples that had been pre-warmed to 25 °C. Samples were incubated at 37 °C for 15 min with constant shaking at 650 rpm (Eppendorf® Thermomixer Compact—T1317). The reaction was stopped by adding EGTA to a final concentration of 2 mM. Fragment size was checked before centrifugation at 400×*g* for 10 min. The pellet was resuspended in salt extraction buffer (250 mM NaCl, 10 mM Tris-HCl pH 7.4, 2 mM MgCl_2_, 2 mM EGTA, 0.1% Triton X-100, with added protease inhibitors) and protein concentration measured by Bradford to take 0.5 mg lysate for ChIP. ChIP was performed as above for U2OS cells with 5 µg of primary antibody and BSA blocked Protein A Sepharose beads. Beads with immunoprecipitated chromatin fragments were then washed three times with low salt washing buffer (20 mM Tris-HCl, pH 8.0, 2 mM EDTA, 150 mM NaCl, 1% Triton X-100, 0.1% SDS, with added protease inhibitors), three times high salt washing buffer (20 mM Tris-HCl, pH 8.0, 2 mM EDTA, 500 mM NaCl, 1% Triton X-100, 0.1% SDS, with added protease inhibitors) and subsequently eluted in 200 μl ChIP elution buffer (20 mM Tris-HCl, pH 8.0, 10 mM EDTA, 1% SDS) for 30 min at 37 °C. DNA was purified using QIAquick PCR purification kit (Cat No./ID: 28106) and quantified using Qubit DNA HS Kit. Ten nanogram of chipped DNA was taken for prepping libraries for next generation sequencing using NEBNext Ultra II library prep kit for Illumina (E7645S). Sequencing was done on a NextSeq500 machine with read length of 76 bases. Following antibodies were used for ChIP; anti-H4K20me1 (Abcam; ab9051—5 µg/sample), anti-H4K20me3 (Abcam; ab9053—7 µg/sample), anti-H4 PAN (Millipore; 04-858—5 µg/sample).

### ATAC-seq

ATAC-seq was performed as originally described by Buenrostro et al.^26,50^ with a few modifications. Briefly, 50,000 cells were washed with 50 µl ice-cold 1× PBS followed by centrifugation at 500×*g* for 5 min. Cells were lysed using cold lysis buffer (10 mM Tris-HCl pH 7.5, 10 mM NaCl, 3 mM MgCl_2_, 0.1% NP40, 0.1% Tween-20 and 0.01% Digitonin). Cells were gently pipetted up and down five times and incubated on ice for 3 min. Five hundred microliter of Wash buffer (10 mM Tris-HCl pH 7.5, 10 mM NaCl, 3 mM MgCl_2_ and 0.1% Tween-20) was added, followed by centrifugation at 500×*g* for 10 min using a refrigerated centrifuge to collect nuclei. The nuclei pellet was resuspended in the transposase reaction mix containing 25 μl of 2×TD buffer, 2.5 μl transposase (Illumina, FC-121-1031), 0.1% Tween-20 and 0.01% Digitonin and nuclease-free water up to 50 µl). The nuclei were resuspended by gently pipetting up and down 6–8 times and incubated at 37 °C for 30 min on thermomixer at 1000 rpm (Eppendorf® Thermomixer Compact—T1317). DNA was then purified using a Qiagen MinElute Kit (Cat No./ID: 28004) in 10 µl of elution buffer. After purification, the DNA fragments were amplified using Nextera PCR master mix (NPM) and 1.25 μM of Nextera PCR Index primers 1 and 2 (Illumina; FC-121-1011), using the following PCR conditions: 72 °C for 5 min; 98 °C for 30 s; and 5 cycles of 98 °C for 10 s, 63 °C for 30 s, and 72 °C for 1 min. We performed the size selection (<600 bp) using Ampure XP magnetic beads (Beckman Coulter Inc.) according to manufacturer’s protocol. To reduce GC and size bias in our PCR, we performed a quantitative real-time PCR (qPCR)-based library quantification. First, one fifth of the purified PCR product was amplified using 2× KAPA SYBR FAST qPCR Master mix (KK4932) for 20 cycles. The optimal number of cycles were determined by the cycle number that corresponds to one third of maximum fluorescent intensity (usually around 6–7 cycles). The full libraries were then amplified for the corresponding number of cycles (determined in previous step) for each sample. The libraries were again purified with size selection (<600 bp) using Ampure XP magnetic beads according to the manufacturer’s protocol. Libraries were quantified using the Qubit DNA HS Kit, and for quality control, 1 μl of each sample was run on Bioanalyzer High Sensitivity DNA Chip. In all, 2 nM of all libraries were pooled and 1.5 pM were sequenced on Illumina NextSeq500 (500/550 High Output v2 Kit—150 cycles) having a read length of 2 × 76 bp (paired-end).

### Microarray

Total RNA was extracted using the Qiagen RNeasy mini extraction kit (Qiagen Sciences, Germantown, MD, USA) kit following the instructions by the manufacturer and quantified using the Nanodrop ND1000 (NanoDrop Technologies, Wilmington, DE, USA). RNA integrities were confirmed with the Agilent Bioanalyzer 2100 using the RNA 6000 Nano Chips (Agilent, Santa Clara, CA, USA). Samples were run on a HT HG-U133+ PM Array Plate (Affymetrix, Santa Clara, CA, USA). Chips were scanned using a GeneTitan microarray scanner and gene expression data (Raw CEL files) were normalized using the Robust Multi‐array Average (RMA) algorithm and the Bioconductor package “Affy” (http://www.bioconductor.org). Differential expression analysis was then done using “limma” package for R (https://www.R-project.org)^51^, followed by GO term enrichment analysis of the differentially expressed genes.

### Sequencing data processing and mapping

The quality of sequenced reads was analysed using FastQC (v. 0.10.1)^52^ and FastqScreen (v. 0.11.4)^53^, and summarized using MultiQC (v. 1.7)^54^. ATAC-seq data were processed and mapped as previously described^4^. Briefly, the raw paired-end reads were first trimmed for Nextera transposase adapter sequences using Trimmomatic (v0.32) in palindrome mode with default settings except ILLUMINACLIP:2:30:10:1:true MINLEN:25. FastQC of reads before and after trimming confirmed the removal of any 3′ adapter sequences, while also clearly showing the known insertion Tn5 motif in the 5′-ends. The trimmed PE reads were mapped to the hg19 assembly (canonical chromosomes only) using bowtie2 v.2.2.9 with default settings except -k 2 -X 2000–no-mixed–nodiscordant. After sorting (SortSam) and labeling duplicates (MarkDuplicates) with Picard tools (v. 2.6.0-27-g915ffa7-SNAPSHOT) and adding a NH tag (number of reported alignments), reads were filtered to exclude unmapped, multimapping, and mitochondrial reads (samtools view -f 2 -F 4 and custom filter). The filtered bam files were converted to bed format using bedtools bamtobed (v2.26.0-92-g88cd6c5), and read start and stop coordinates were finally adjusted by +5 bp and −4 bp, respectively, to adjust for Tn5-binding properties as previously described^26^. ChIP-seq reads were mapped to canonical chromosomes from mm10 or hg19 using Bowtie (v. 1.1.2)^55^ using the -m 1 setting to avoid reads mapping to multiple locations. Output was converted to bed files using Samtools (v. 1.10)^56^, and BedTools (v. 2.26.0) bamToBed^57^. Signal quality was evaluated using the Phantom peak algorithm (https://github.com/kundajelab/phantompeakqualtools)^58^.

### Integrative analysis of ChIP-seq, ATAC-seq, and gene expression data

Mapped ATAC-seq and ChIP-seq data were imported in to EaSeq (v. 1.111)^59^ as “Datasets”. Genomic loci of interest as well as genome-wide expression data were imported into EaSeq as “Regionsets”, and when nothing else is mentioned subsequent visualization was done using EaSeq and integrated tools. To visualize signal at genes, multiple entries with the same gene symbol were collapsed so that each gene symbol was only represented once and the outermost coordinates were used. A list of unique TSSes was obtained using the “Extract”-tool and redundant coordinates removed using “Regionset/Modify”-tool and the settings “Merge with…” and selecting the TSS coordinates both as source and target “Regionset”. Ten kilobasepair windows were obtained by importing the chromosome sizes for each chromosome in the reference genome as a “Regionset” and using “Regionset/Modify”-tool and the settings “Homogenize and fragment regions to” and “10,000” bp. For genes and 10 kbp windows, values were quantified using the “Quantify”-tool and default settings (resulting in values being normalized to FPKMs, Fragments per Kilobasepairs per Million reads), except that start and end positions were set to region “Start” and “End”, respectively, and offsets set to “0” bp. For TSSes and enhancers, values were quantified using the “Quantify”-tool and default settings (Resulting in FPKM normalized values), except that start and end offsets were set to “−1000” and “1000” bp, respectively. Ten kilobasepair windows with very few or no reads due to low “mappability” were excluded from downstream visualization and analysis. ATAC-seq data quantified in 10 kbp windows were quantile normalized using the “Normalize”-tool, and values for MA-plots were derived by averaging normalized values from all biological replicates, and calculating the M component as log_2_(SET8-kd/Ctrl-kd) and the A component as 0.5 * log_2_(SET8-kd * Ctrl-kd) from the averaged quantile normalized values. To obtain subsets of windows overlapping with genes or not, the “Coloc.”-tool was used with the setting “Border to border of the regions” followed by gating out subsets with border distances =<0 or >0 for overlapping, and non-overlapping regions, respectively. A similar strategy was used to obtain subsets of windows overlapping with house-keeping genes or not. Statistical analyses were performed using R (https://www.R-project.org)^51^ and simple calculations and database functions were performed in Microsoft Excel.

### Visualization of ATAC-seq, ChIP-seq, and expression data

When nothing else is stated, visualization was done using EaSeq (v. 1.111)^59^ and integrated tools and exported for layouting using the “Export snapshot as pdf” tool in the Beta-testing panel. Color scales were developed by Smith and van der Walt as well as Harrower and Brewer^60^ (see https://bids.github.io/colormap/ and http://www.colorbrewer.org/). 2D-histograms were obtained using the “Scatter”-tool, and colored 2D-histograms were obtained using the “ZScatter”-tool with the number of bins on the axes set to 100 and “Count” adjusted as indicated in the “Plot Settings”. Graphs of the average distribution of signal along gene bodies were obtained using the “Av. Track”-tool followed by selecting the relevant graphs and overlaying them using the “Overlay”-tool. Box plots were obtained using R (https://www.R-project.org)^51^, simple vertical 1D heatmaps of numerical values were generated using the “ParMap”-tool, and Heatmaps of ChIP-seq signal distribution along several loci were generated using the “HeatMap” (for heatmaps of one ChIP-seq data set), “RatioMap” (for heatmaps of ratios between two ChIP-seq datasets), and “NSMap” (for heatmaps showing the average signal from multiple loci with the same properties, e.g., gene sizes) tools with plot settings adjusted as indicated in the figures. For heatmaps and tracks depicting gene bodies as well as upstream and downstream regions, the “Plot Settings” were adjusted to relative sizes (“Rel.” flag checked) and “Window size” set to 200%. Meta-gene plots of gene quartiles were generated by subdividing genes into four equal sized groups according to average expression in control siRNA-treated synchronized unreleased U2OS cells. The average signal within each group was plotted for each replicate using the “Av. Track” tool, and these were overlaid using the “Overlay”-tool and underlying values exported for calculation of mean and standard deviations using the “Export values from plots” tool in the Beta-testing panel. Violin plots were generated using the “Histogram”-tool. Pearson’s correlation coefficients in 2D-histograms of NGS data generally resulted in *p*-values at the lowest possible number (*p* < 2.2e−16) that can be obtained using the “cor.test()” in R (https://www.R-project.org)^51^.

### Clustering of genes based on ChIP-seq data and integration of expression data

k-means clustering of genes according to H4K20me1 levels was done using the ‘Cluster’-tool with the following settings: No log-transformation, k-means, Start position = start, End position = end, Start offset = 0, End offset = 0. For MEF data the clustered set of regions consisted non-redundant genes (see above), for U2OS data the clustered set of regions came from the microarray expression analysis output. For MEF ChIP-seq data, the following datasets were used for the clustering: Ctrl-kd H4K20me1, Ctrl-kd input, SET8-kd H4K20me1, and SET8-kd input. For U2OS ChIP-seq data, the following H4K20me1 datasets were used for the clustering: Ctrl-kd 0 h, Ctrl-kd 8 h, Ctrl-kd 16 h, SET8kd 0 h, SET8-kd 8 h, and SET8kd 16 h.

The number of genes within each cluster defined by U2OS H4K20me1 levels were counted and the number of observed genes, which were significantly upregulated or downregulated at the 16 h time point based on adjusted *p*-values, was counted. This was related to the expected counts based on the cluster sizes and general frequency of upregulated or downregulated genes, and the log_2_-fold difference was used to color the bubbles in the bubble diagram. Significance levels were obtained using chi-square tests on these numbers Benjamini–Hochberg corrected for multiple testing, and the –log_10_ values from this output used to determine bubble sizes.

### Overexpression and purification of histones

*Homo sapiens* histones H2A, H2B, H3, and H4 were overexpressed and purified following published protocols^23,61^. The pET-3a plasmids containing the histone sequences were transformed and overexpressed in E. *coli* BL21 (DE3) pLysS cells in 2 TY media. Crude WT histones were purified by 26/60 Sephacryl S-200 column (GE Healthcare) followed by the Resource S cation-exchange column (GE Healthcare). The expression of H4K20C mutant was similar to that of WT histones. H4K20C histone purified by gel-filtration (Sephacryl S-200 column) was further purified by Vydac C4 preparative HPLC chromatography (Grace).

To produce ^13^C-labeled,^15^N-labeled histones, cells were grown in 2x TY media until OD_600_ reached 0.5. The cells were pelleted down and resuspended in equal volume of M9 minimal media supplemented with 0.4% ^13^C glucose and 0.1% ^15^N ammonium chloride (CIL, USA), micro nutrients and BMEvitamins (Sigma-Aldrich). After 1 h acclimatization, the cells were induced with 0.4 mM IPTG at OD_600_ of 0.7 for overexpression for 3 h at 37 °C. The ^13^C-labeled,^15^N-labeled WT H4 and H4K20C were purified following the same procedures as the unlabeled ones.

### Installation of methyl-lysine analogs

To generate histone H4 with mono-methylation and tri-methylation on Lys20, we followed the modified published protocol, which results in the replacement of the lysine side chain with the methylated analog that has an S atom at the gamma position (denoted K_C_20 methylation)^37^. Previous in vitro investigations established that the K_C_20 methylation analogs behave similarly to the native counterparts with respect to biochemical and biophysical properties^37,41^.

For monomethylation installation, briefly, 10 mg of pure H4K20C protein was dissolved in 900 μL alkylation buffer (6 M Gdn·HCl, 1 M HEPES, 10 mM D/L methionine, pH 7.8), and incubated in 37 °C for 1 h after the addition of 20 μL of freshly prepared 1 M Dithiothreitol (DTT). Thirteen milligram of (2-chloroethyl)methylammonium chloride was added, and the reaction took place for 4 h at room temperature. Another 10 μL of 1 M DTT and 2.6 mg of (2-chloroethyl)methylammonium chloride was added, and the reaction proceeded overnight at room temperature.

For trimethylation installation, briefly, 40 mg of H4K20C protein was dissolved in 4.9 mL of alkylation buffer. Hundred microliter of freshly prepared 1 M DTT was added to the solution. After 10 min, 300 mg of (2-bromoethyl)trimethylammonium bromide was added. The alkylation reaction took place at 50 °C. After 5 h, another 50 μL of 1 M DTT and 100 mg of (2-bromoethyl)trimethylammonium bromide was added. The reaction proceeded for another 3 h at 50 °C.

For both mono-methylation and tri-methylation, the reaction was quenched with Trifluoroacetic acid (TFA), after which the mixture was dialyzed against water (containing 0.1% TFA) overnight. The final product was obtained after lyophilization. The molecular weight of the final product was confirmed by MALDI-TOF MS.

### Histone octamer refolding, DNA preparation, and chromatin preparation

Histones (hH2A:hH2B:hH3:H4) were mixed in unfolding buffer (7 M Gdn∙HCl, 10 mM Tris·HCl, 10 mM DTT, pH 7.5) at molar ratio of 1.2:1.2:1:1 and dialyzed against refolding buffer (10 mM Tris⋅HCl, 2 M NaCl, 1 mM EDTA, 5 mM beta-mercaptoethanol, pH 7.5) overnight. The histone octamer (HO) was purified by gel filtration chromatography using HiLoad 16/600 Superdex 200 pg column (GE Healthcare), and fractions were checked with 18% SDS PAGE.

The 15-601-197 DNA (15 repeats of the Widom’s “601” high affinity nucleosome positioning sequence with 197 bp NRL) originally constructed in pUC18 vector from Prof. Daniela Rhodes laboratory was subcloned to pWM530 vector. Amplified plasmid was digested by EcoRV, followed by PEG6000 fractionation. Desired DNA fragment was further purified by HiPrep 26/60 Sephacryl S-500 HR column (GE Healthcare). The purity of the fractions was checked by 0.7% agarose gel.

The reconstitution of 15-197 nucleosome arrays was conducted following published protocols^20,23^. 15-197 DNA, purified HOs, and 147-bp competitor DNA were mixed and salt gradient dialysis took place at 4 °C overnight in TEN 2.0 buffer (10 mM Tris·HCl, 2 M NaCl, and 0.1 mM EDTA, pH 7.5) to TEN 0.01 buffer (10 mM Tris·HCl, 10 mM NaCl, and 0.1 mM EDTA, pH 7.5). The mass ratio of 15-197 DNA and 147-bp competitor DNA was 1:0.05 for all reconstitutions. The array solution was centrifuged at 20,000×*g* for 10 min after dialysis. The saturation point of reconstituted arrays was determined by electrophoresis in 0.8–1.1% agarose gel and checked by analytical ultracentrifugation. 16-187-601 and 12-202-601 DNA and nucleosome arrays were prepared in the same way as 15-197-601 constructs except that for the DNA preparations, amplified pUC18 plasmids containing 16-187-601 or 12-202-601 DNA were digested with EcoRV, HaeII, DdeI and DpnI.

### AUC-SV

AUC-SV measurements were conducted using a Beckman XL-A/XL-I analytical ultracentrifuge (Beckman Coulter, Brea, CA) with an 8-hole An-50 Ti analytical rotor as described in the previous work^23,24^. The nucleosome arrays were diluted in TEN 0.01 buffer to optical density *A*_259_ = 0.6–0.8. A single scan at 3000 rpm (655 g at cell center) was performed after equilibration under vacuum at 20 °C for 2 h. Then 40 scans were collected at 12,000 rpm (10,483 g at cell center) with a 10-min interval. The software Ultrascan III was employed for data analysis using the enhanced van Holde–Weischet method. The sedimentation velocity coefficient was corrected to s_20,w_ using a partial specific volume of 0.622 mL/g. Two to three independent measurements were performed for each array sample at each designated salt concentration. The mean s_20,w_ value was calculated from s_20,w_ (obtained from 20 to 80% of the boundary) of 2–3 experiments with the SD obtained as the RMS of the s_20,w_. Graphs were made using Graphpad Prism 8 (8.4.3).

### Precipitation assay

Reconstituted 15-197 array was diluted in 10 mM Tris⋅HCl (pH 7.5) to an optical density A_260_ = 2. The array solution (10 μL) was mixed with equal volume of buffer containing twice the final concentration of MgCl_2_. After incubation at room temperature for 15 min, the samples were centrifuged at 20,000×*g* for 15 min at 20 °C. The absorbance of the supernatant was measured at 260 nm using NanoDrop ND-1000 UV/Vis spectrophotometer (NanoDrop Technologies, Wilmington, DE, USA). The efficiency of Mg^2+^ to precipitate the array (EC_50_) was characterized by the Mg^2+^ concentration at 50% precipitation of the array (EC_50_) after applying sigmoidal model fitting.

### NMR sample preparation

Reconstituted 15-197 arrays were concentrated with an Amicon concentrator device (MWCO: 10 kDa, Millipore) to 8–9 mg/mL, and treated with 5 mM MgCl_2_ (final concentration). The precipitated samples were transferred into a rotor packing device (Giotto Biotech) assembled with a 1.9 mm SSNMR rotor. Ultracentrifugation at 100,000×*g* was performed for 5–8 h to bring down the nucleosome array aggregates into the 1.9 mm SSNMR rotor.

### Solid-state NMR experiments

All ssNMR experiments were conducted with an 18.8T Bruker Advance III HD spectrometer equipped with a 1.9 mm HCN magic angle spinning (MAS) probe. The sample temperature was maintained at 11–13 °C, which was calibrated externally with ethylene-glycol^62^. The ^13^C chemical shift was referenced at the DSS scale using adamantine with the downfield signal set at 40.48 ppm. The ^1^H and ^15^N chemical shifts were indirectly calculated based on the ^13^C reference ^63^. All experiments were performed with a MAS frequency of 17857 Hz. The 90° pulse lengths of ^1^H, ^13^C, and ^15^N were 2.4 μs, 3.2 μs, and 4.15 μs, respectively. In the 2D J-coupled ^1^H-^13^C correlation experiments, the spectral width and acquisition time were 266.25 ppm and 19.1 ms in the direct dimension, and 11.16 ppm and 15.2 ms in the indirect dimension. In the 2D J-coupled ^1^H-^15^N correlation experiments, the spectral width and acquisition time were 220.22 ppm and 19.7 ms in the direct dimension, and 3.72 ppm and 16.1 ms in the indirect dimension. The spectral width and acquisition time used in the 2D ^13^C–^13^C DARR experiments were 266.23 ppm and 15.5 ms in the direct dimension and 266.23 ppm and 8.4 ms in the indirect dimension. In the 2D NCA experiments, the spectral width and acquisition time were 266.25 ppm and 14.4 ms in the direct dimension, and 44.0 ppm and 12.3 ms in the indirect dimension. In the 2D NCO experiments, the spectral width and acquisition time were 88.74 ppm and 14.4 ms in the direct dimension and 36.7 ppm and 12.1 ms in the indirect dimension. The NCA and NCO transfer was achieved by SPECIFIC-CP^64^. In all experiments, the recycle delay was set as 1.5 s. All ssNMR data was processed using nmrPipe^65^. and analyzed with Sparky (T. D. Goddard and D. G. Kneller, University of California, San Francisco).

## Supplementary information

Supplementary Information

## Data Availability

The data that support this study are available from the corresponding authors upon reasonable request. The ChIP-seq data generated for this publication have been deposited in NCBI’s Gene Expression Omnibus^66^ and are accessible through GEO Series accession number GSE156216. ATAC-seq data from asynchronous cells, ATAC-seq data from the 0 h (t0), and from the 8 h (t8) time points are accessible through GEO Series accession number GSE156215 and ATAC-seq data from DRB-treated cells and controls are available through GEO Series accession number GSE173313. ATAC-seq data from the 16 h time point were previously published^4^ and are available at are accessible through GEO Series accession number GSE118606 . Previously published H3K9me3 and input data^67^ were downloaded from the NCBI GEO database^66^ from GEO Series accession number GSE31755. Refseq gene annotations^68^ were acquired from the UCSC table browser^69^. Enhancers were obtained from the Fantom 5 consortium^70^, and a list of human house-keeping genes was obtained from^31^. The mass spectrometry proteomics data have been deposited to the ProteomeXchange Consortium via the PRIDE^71^ partner repository with the dataset identifier PXD027150. Source data are provided with this paper.

## Source data

Source Data
