# Supplementary material for: Histone H4 lysine 20 mono-methylation directly facilitates chromatin openness and promotes transcription of housekeeping genes
Source: Nat Commun. 2021 Aug 20;12:4800. doi: 10.1038/s41467-021-25051-2 (PMC8379281; doi:10.1038/s41467-021-25051-2)
Supplement: Supplementary file 1 — Supplementary Information [file 41467_2021_25051_MOESM1_ESM.docx]

Supplementary information

**Histone H4 lysine 20 mono-methylation directly facilitates chromatin openness and promotes transcription of housekeeping genes**

Shoaib, et al


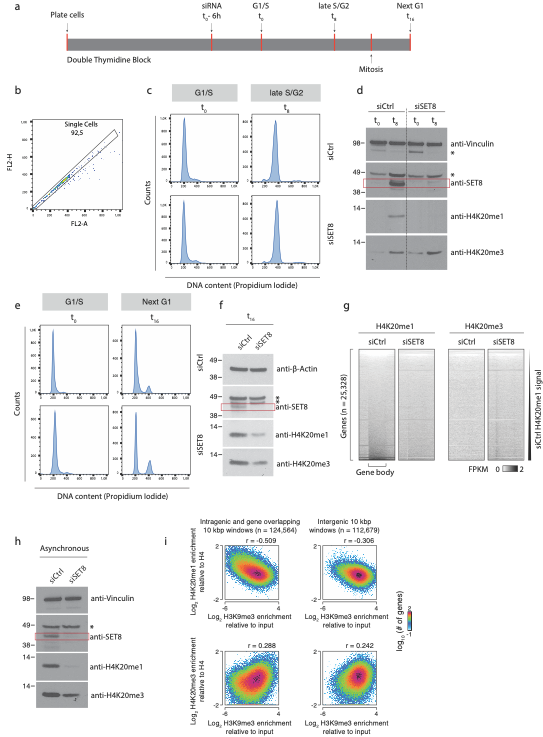


**Supplementary Fig. 1.**

(a) Schematic presentation of the experimental design. U2OS cells were synchronized with double thymidine block. Cells were either treated with control or SET8 siRNA 6 hours before release (t0 -6 h). Samples were then collected at the time of release (G1/S – t0), at 8 hours after the release (late S/G2 – t8) or 16 hours after the release (next G1 – t16). (b) Single cell gating strategy for flow cytometry profiles in (c) and (e). Briefly, FL2-A vs FL2-H was used to gate for single cells. The cell cycle profile shown in (c) and (e) represents the cell population from this single cell gate (c) Flow cytometry profiles of samples from (a) corresponding to G1/S (t0) and late S/G2 (t8) phases of cell cycle. (d) Immunoblotting of samples from (b) with indicated antibodies. Immunoblots were repeated for 2 replicates with consistent results. * represents unspecific band. SET8-specific band is marked by a red rectangular outline (e) Flow cytometry profiles of samples from (a) corresponding to G1/S (t0) and next G1 (t16) phases of cell cycle. (f) Immunoblotting of samples from (e) with indicated antibodies. Immunoblots were repeated for 3 replicates with consistent results. * represents unspecific band. SET8-specific band is marked by a red rectangular outline (g) Heatmaps of H4K20me1 and input levels at human genes in SET8-kd and control-kd U2OS cells at G1/S (t0). Genes were ordered according to average H4K20me1 in control-kd U2OS cells in the gene body. The horizontal extent of each gene and the upstream and downstream regions corresponding to half a gene length is fitted within the same visual space in the heatmaps regardless of its absolute extent. H4K20me1 and input levels are FPKM-normalized. (h) Immunoblotting of samples from asynchronously growing U2OS cells treated with either control or SET8 siRNA for 48 hours with indicated antibodies. Immunoblots were repeated for 3 replicates with consistent results. * represents unspecific band. SET8-specific band is marked by a red rectangular outline (i) 2D-histograms showing the genome-wide relationships between enrichment of H3K9me3 levels (X-axes) and of selected H4K20 methylation states (Y-axes) and in 10 kbp windows within or overlapping with genes (left side) or outside of genes (right side). Y-axes shows log2 normalized H4K20me1 (top) or H4K20me3 (bottom) ChIP-seq levels relative to the levels of H4 control ChIP-seq, while Y-axes shows log2-normalized H3K9me3 levels relative to the levels in corresponding input samples from U2OS cells. H4K20 methylation data were generated from cells synchronized and harvested in G1/S (0 hrs), while H3K9me3 data were from a published external source (see methods). The colour scales reflect the number of genes having a certain combination of H4K20me and H3K9me3 enrichment. r-values show the Pearson’s correlation coefficients for the data in each plot. Source data are provided as a Source Data file.

**Supplementary** **Figure 2.**

(a) 2D-histograms showing the relationship between enrichment of selected H4K20 methylation states (X-axes) and chromatin accessibility probed using ATAC-seq (Y-axis) at human enhancers. X-axes shows log_2_ normalized H4K20me1 (left side) or H4K20me3 (right side) ChIP-seq levels relative to the levels of H4 control ChIP-seq, while Y-axes shows log_10_-transformed average FPKM-normalized ATAC-seq signal from synchronized U2OS cells 0 hrs (top, three replicates), 8 hrs (middle, three replicates) and 16 hrs (bottom, four replicates) after release. The colour scales reflect the number of genes having a certain combination of K20me enrichment and ATAC-seq. r-values shows the Pearson’s correlation coefficients for the data in each plot. (b) 2D-histograms showing the relationship between enrichment of selected H4K20 methylation states (X-axes) and chromatin accessibility probed using ATAC-seq (Y-axis) at human enhancers as in a. Y-axes shows log_10_-transformed average FPKM-normalized ATAC-seq signal from four replicates of U2OS cells grown asynchronously. (c) 2D-histograms showing the relationship between enrichment of selected H4K20 methylation states (X-axes) and chromatin accessibility probed using ATAC-seq (Y-axis) at human TSSes. X-axes shows log_2_ normalized H4K20me1 (left side) or H4K20me3 (right side) ChIP-seq levels relative to the levels of H4 control ChIP-seq, while Y-axes show log_10_-transformed average FPKM-normalized ATAC-seq signal from synchronized U2OS cells 0 hrs (top, three replicates), 8 hrs (middle, three replicates) and 16 hrs (bottom, four replicates) after release. The colour scales reflect the number of genes having a certain combination of K20me enrichment and ATAC-seq. r-values shows the Pearson’s correlation coefficients for the data in each plot. (d) 2D-histograms showing the relationship between enrichment of selected H4K20 methylation states (X-axes) and chromatin accessibility probed using ATAC-seq (Y-axis) at human TSSes as in c. Y-axes shows log_10_-transformed average FPKM-normalized ATAC-seq signal from four replicates of U2OS cells grown asynchronously.

**Supplementary** **Figure 3.**

(a) Box plots showing the relationship between differently sized genes (X-axis; subdivided into four equal sized groups each containing 6332 genes) and H4K20me1 (left panel) or H4K20me3 (right panel) ChIP-seq signal normalized to H4 control ChIP-seq. Boxes depict the interquartile range, the belt depicts the median, and whiskers depict the most extreme sample values or 1.5 x the interquartile range depending on which of these values that are closest to the median. (b) 2D-histograms showing the relationship between enrichment of selected H4K20methylation states (X-axes) and genes sizes (Y-axis). X-axes show log_2_ normalized H4K20me1 (left side) or H4K20me3 (right side) ChIP-seq levels relative to the levels of H4 control-kd ChIP-seq, while Y-axes shows log_10_ transformed gene lengths. The colour scales reflect the number of genes having a certain combination of K20me enrichment and gene length. r-values shows the Pearson’s correlation coefficients for the data in each plot. (c) Heatmaps showing the distribution of H4K20me1 signal at genes sorted according to their size (Y-axis). The X-axes shows the average normalized signal distribution for all genes in the group with this particular size in the area ranging from -10kbp to +100kbp of the TSS. H4K20me1 signal was obtained in control siRNA treated and synchronized U2OS cells without release and was denominated as FPKMs. Leftmost plots: schematic illustration with dotted lines to indicate the positions of transcription start sites (TSS, green) and transcription termination sites (TTS, orange) in the plots. Three different subsets of coordinates were used for the plot: all transcripts annotated in RefSeq (Left), a collapsed set of genes where each gene symbol is represented once and the outermost set of coordinates were used (middle), and the subset of these which were at least 10 kbp apart from their nearest neighbour gene (right). (d-e) 2D-histograms showing the relationship between gene size (d, X-axis) or average ATAC-seq density in each gene (e, X-axis) and enrichment of H4K20me1 (Y-axes). Y-axes show log_2_ normalized H4K20me1 ChIP-seq levels relative to the levels of H4 control-kd ChIP-seq. The colour scales reflect the number of genes having a certain combination of K20me enrichment and gene length. r-values shows the Spearman’s ranked correlation coefficients for the data in each plot. (f) Box plot illustrating the enrichment of H4K20me1 (Y-axis) at genes subdivided into five equal sized groups (each containing 5065 genes) based on ranked genes size (blue, inverted: leftmost group contain the largest genes) or ATAC-seq signal (orange, first group contain the least accessible genes). Y-axis show log_2_ normalized H4K20me1 ChIP-seq levels relative to the levels of H4 obtained in control siRNA treated and synchronized U2OS cells without release. P-values were calculated by comparing each of the five subgroups to its counterpart using Mann-Whitney U-tests (two-sided test) and Bonferroni corrected for multiple testing. Boxes depict the interquartile range, the belt depicts the median, and whiskers depict the most extreme sample values or 1.5 x the interquartile range depending on which of these values that are closest to the median.

**
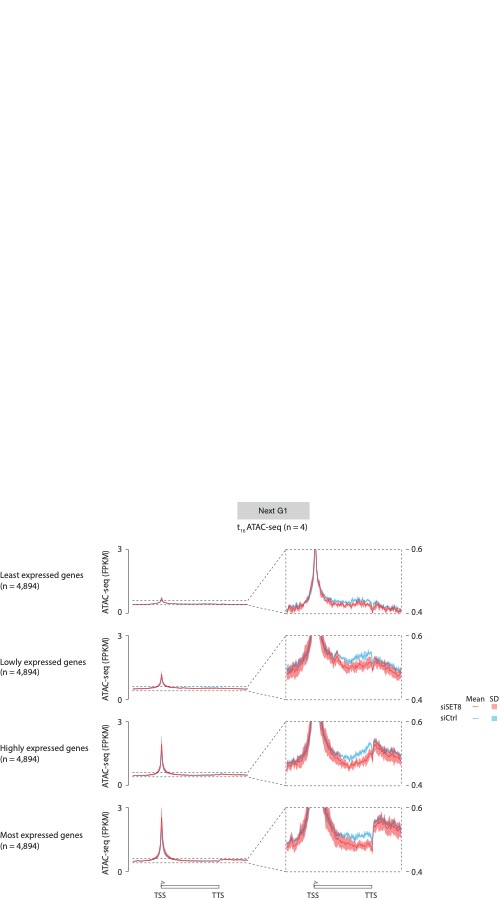
**

**Supplementary Fig. 4.**

Metagene plots showing the mean and mean +/-1 standard deviation (SD) ATAC-seq signal intensity in synchronized U2OS cells 16 hours after the release (next G1 – t16). X-axis location depicts the signal from start to end of each gene body including areas corresponding to 50% gene length upstream TSS and downstream TTS. Thus, the horizontal extent of each gene and the upstream and downstream regions corresponding to half a gene length is fitted within the same visual space in the heatmaps regardless of its absolute extent. Cells were either treated with control siRNA (four replicates) or SET8 siRNA (four replicates) and FPKM-normalized ATAC-seq values quantified at genes divided into quartiles depending on expression. Mean and standard deviation were calculated from the average of all genes within each quartile based on the four replicates in each sample.

**
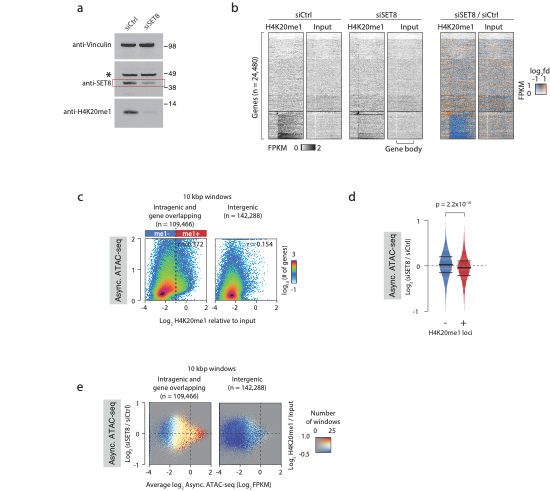
**

**Supplementary Fig. 5.**

(a) Asynchronously growing mouse embryonic fibroblasts were treated with indicated siRNAs, harvested after 72 h and blotted with indicated antibodies. * represents unspecific band. SET8-specific band is marked by a red rectangular outline. Immunoblots were repeated for 3 replicates with consistent results (b) 2D-histograms showing the relationship between enrichment of H4K20me1 (X-axes) and chromatin accessibility probed using ATAC-seq (Y-axis) measured in 10kbp windows and shown separately for windows overlapping fully or partially with annotated gene bodies (left) and the windows that did not overlap (right). Y-axes show average FPKM-normalized ATAC-seq signal from four replicates of MEF cells grown asynchronously. The dashed line and markings above the left plot show the threshold selected for scoring windows as H4K20me1-negative (blue) or -positive (red). (c) Coloured 2D-histograms showing the genome-wide three-way relationship between enrichment of H4K20me1 (colour) and the level of ATAC-seq signal in SET8-kd and control-kd MEF cells. ChIP-seq and ATAC-seq signal was measured in 10kbp windows and shown separately for windows overlapping fully or partially with annotated gene bodies (left) and the windows that did not overlap (right). ATAC-seq signal is plotted as MA-plots with average log_2_ signal from the two conditions on the X-axis and the log_2_ difference between SET8-kd and control-kd on the Y-axis. Colouring shows log_2_ normalized H4K20me1 ChIP-seq levels relative to the input as indicated with the number of windows with a certain combination of ATAC-seq levels (X-axis) and change (Y-axis) controlling opacity as indicated in the right-side colour scale. Plots show FPKM-normalized ATAC-seq signal from MEF cells grown asynchronously (four replicates). (d) Violin plot of log_2_ differences in ATAC-seq signal in SET8-kd and control-kd MEF cells for the H4K20me1–negative and –positive windows scored as illustrated in (b). Horizontal lines depict the 25, 50, and 75 percentiles, and the p-value is obtained using a Mann-Whitney U-test (two-sided test) (p=2.2x10-16). (e) Heatmaps of mouse genes clustered using k-means in accordance with H4K20me1 and input distributions in SET8-kd and control-kd MEF cells from start to end of each gene body. The horizontal extent of each gene and the upstream and downstream regions corresponding to half a gene length is fitted within the same visual space in the heatmaps regardless of its absolute extent. Left and middle heatmaps show FPKM-normalized H4K20me1 ChIP-seq (left) or input signal (right), whereas right side heatmaps are color-coded and shows the ratio between signals from SET8-kd and control-kd MEF cells with increased, unchanged, and decreased levels being depicted as orange, black, and blue, respectively. The opacity is adjusted to the maximum signal from either of the compared conditions. Source data are provided as source data file.


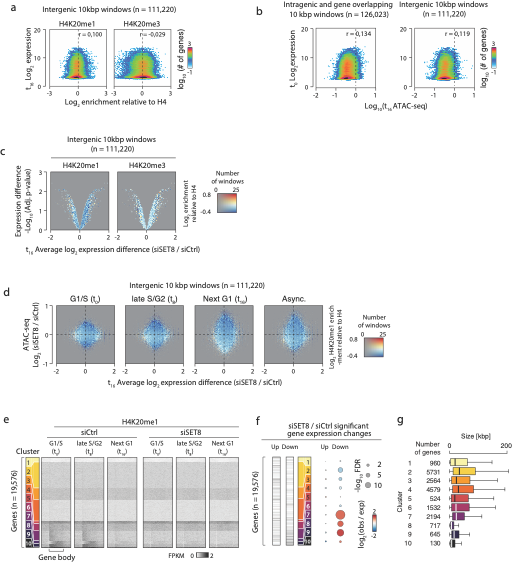


**Supplementary Fig. 6.**

(a) 2D-histograms showing the relationship between enrichment of selected H4K20methylation states (X-axes) and expression assayed using microarrays (Y-axis) at intergenic loci. X-axes shows log2 normalized H4K20me1 (left side) or H4K20me3 (right side) ChIP-seq levels relative to the levels of H4 control ChIP-seq, while Y-axes shows log2-transformed expression levels from synchronized U2OS cells 0 hrs after release. Signal was quantified in 10 kbp windows and the subset not overlapping fully or partially with any annotated gene body was visualized. The colour scales reflect the number of windows having a certain combination of K20me enrichment and expression. r-values shows the Pearson’s correlation coefficients for the data in each plot. (b) 2D-histograms showing the relationship between ATAC-seq signal (X-axes) and expression assayed using microarrays (Y-axis) at intergenic loci. X-axes shows log10 FPKM normalized ATAC-seq signal from four replicates of synchronized U2OS cells 16 hrs after release, while Y-axes shows log2-transformed expression levels from synchronized U2OS cells 0 hrs after release. Signal was quantified in 10 kbp windows and the subset overlapping fully or partially with any annotated gene body was visualized in the left plot whereas the remained was visualised in the right plot. The colour scales reflect the number of windows having a certain combination of ATAC-seq signal and expression. r-values shows the Pearson’s correlation coefficients for the data in each plot. (c) Coloured volcano plots showing the three-way relationship between enrichment of selected H4K20methylation states (colour) within intergenic 10kbp windows, and the expression difference of the nearest gene (X-axis), and significance of the expression difference of the nearest gene (Y-axis). Colouring shows log_2_ normalized H4K20me1 (left side) or H4K20me3 (right side) ChIP-seq levels relative to the levels of H4 control ChIP-seq opacity is controlled by the number of genes having the combination of X- and Y-axis values as indicated in the right side colour scale. X-axes shows log_2_-transformed difference between SET8 knockdown and siRNA controls, and Y-axes shows –log_10_ adjusted p-values from the analysis of the differential expression from two replicates of SET8 knockdown and three replicates of siRNA control synchronized U2OS cells 16 hrs after release. (d) Coloured 2D-histograms showing the genome-wide three-way relationship between enrichment of H4K20me1 (colour), expression changes of the nearest gene (X-axis), and ATAC-seq signal changes in SET8-kd and control-kd U2OS. ChIP-seq and ATAC-seq signal was measured in 10kbp windows and the subsets not overlapping with any annotated gene body. ATAC-seq signal and expression differences are plotted the log_2_ difference between SET8 knockdown and siRNA controls. Colouring shows the average log_2_ normalized H4K20me1 ChIP-seq levels relative to the levels of H4 control ChIP-seq with the number of windows with a certain combination of expression change (Y-axis) and ATAC-seq change (X-axis) controlling opacity as indicated in the right-side colour scale. Plots shows FPKM-normalized ATAC-seq signal from separate rounds of experiments as follows: synchronized U2OS cells 0 hrs (leftmost, three replicates), 8 hrs (middle left, three replicates) and 16 hrs (middle right, four replicates) after release, or U2OS cells grown asynchronously (rightmost, four replicates). (e) Heatmaps showing H4K20me1 signal at human genes clustered according to H4K20me1 distribution within the gene bodies in U2OS cells treated with siSET8 and siCtrl. Signal is normalized as FPKMs. Gene bodies are adapted to take up the same visual space regardless of their absolute size in bp, and the horizontal axis in all plots shows the gene bodies as well as upstream and downstream areas corresponding to 50% of gene length. (f) Heatmaps and bubble plot illustrating the expression changes in genes clustered according to H4K20me1 distribution as in (e). The heatmaps are vertically ordered as in (e) and illustrate the density of significantly up- (left) or down-regulated (right) genes. For each cluster the number of significantly up- and downregulated genes was counted and compared to the expected count if all clusters had equally frequent up- and down-regulated genes using χ^2^-testing Benjamini-Hochberg corrected for multiple testing. Bubble colours illustrate the log2 fold difference between observed and expected gene counts and bubble sizes the –log10 adjusted p-values. (g) Box plot showing the size distribution of genes within each cluster. Clusters correspond to those used in (e-f). Boxes depict the interquartile range, the belt depicts the median, and whiskers depict the most extreme sample values or 1.5 x the interquartile range depending on which of these values that are closest to the median.

**
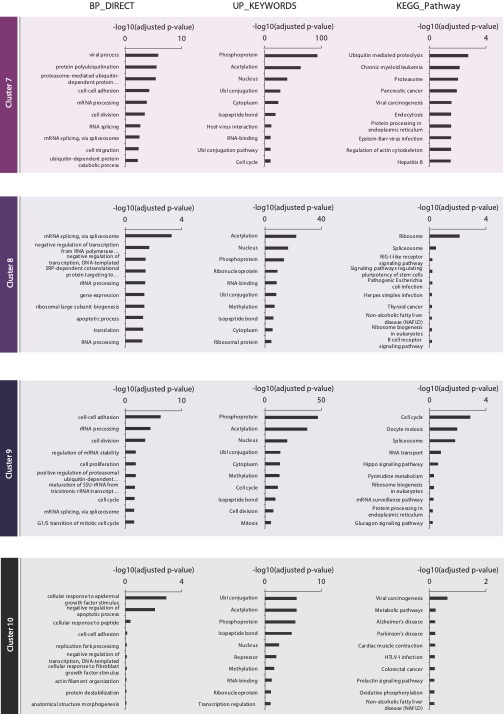
**

**Supplementary Fig. 7.**

Gene ontology analysis of clusters 7-10 from Supplemental fig. 6e-g.

**Supplementary Fig. 8.**

(a) U2OS cells were transfected with either control siRNA or SET8 siRNA for 36 h and treated with transcriptional inhibitor DRB (5,6-Dichloro-1-beta-Ribo-furanosyl Benzimidazole) (100 µM) for 2 h prior to harvesting the cells. For nascent RNA labeling, U2OS cells were pulse labelled with 5-ethynyl uridine (5-EU) (5 mM) for 1 h before fixation. (b) Bar plot showing mean values for 5-EU in the four conditions.

**
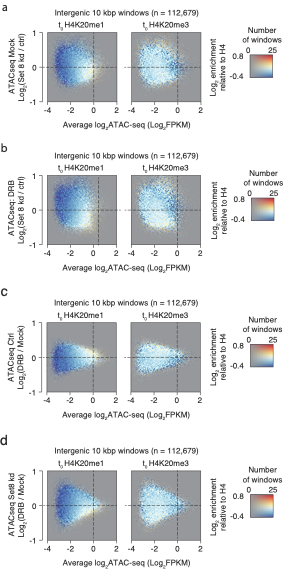
**

**Supplementary Fig. 9.**

(a-d) Coloured 2D-histograms showing the genome-wide relationship between enrichment of H4K20methylation enrichment (colour) and the changes in ATAC-seq signal as a consequence of SET8-knockdown in untreated (a) or DRB-treated (b) U2OS cells as well as changes due to DRB-treatment in control (c) or SET8 knockdown (d) U2OS cells. ChIP-seq and ATAC-seq signal was measured in 10kbp windows and shown for windows not overlapping with annotated gene bodies. ATAC-seq signal is plotted as MA-plots with average log2 signal from the two conditions on the X-axis and the log2 difference between the conditions on the Y-axis. Colouring shows log2 normalized H4K20me1 (right plot) or H4K20me3 (left plot) ChIP-seq levels relative to the levels of H4 control ChIP-seq as indicated, with the number of windows with a certain combination of ATAC-seq levels (X-axis) and change (Y-axis) controlling opacity as indicated in the right side colour scale. Plots shows averaged FPKM-normalized ATAC-seq signal from our replicates in each condition of U2OS cells grown asynchronously.

**Supplemental Fig. 10.**

(a) 15-197-601 and 16-187-601 nucleosome arrays reconstituted with different human histone constructs analyzed by 1.1% agarose gel at the saturation point corresponding to 15 nucleosomes per 15-197 bp DNA array template and 16 nucleosomes per 16-187 bp DNA template respectively. (b-d) Representative van Holde-Weischet curves. The boundary fraction versus s_20,w_ for 15-197 (b, c) and 16-187 (d) arrays at varying salt concentrations for the different histone H4K20 methylation constructs in the presence of MgCl_2_ and NaCl, is displayed. For all constructs, two to three independent measurements were performed and the average s_20,w_ obtained from the 20–80% range of the boundary resulted in the values displayed in Figure 5b and e. Source data are provided as a Source Data file.

**
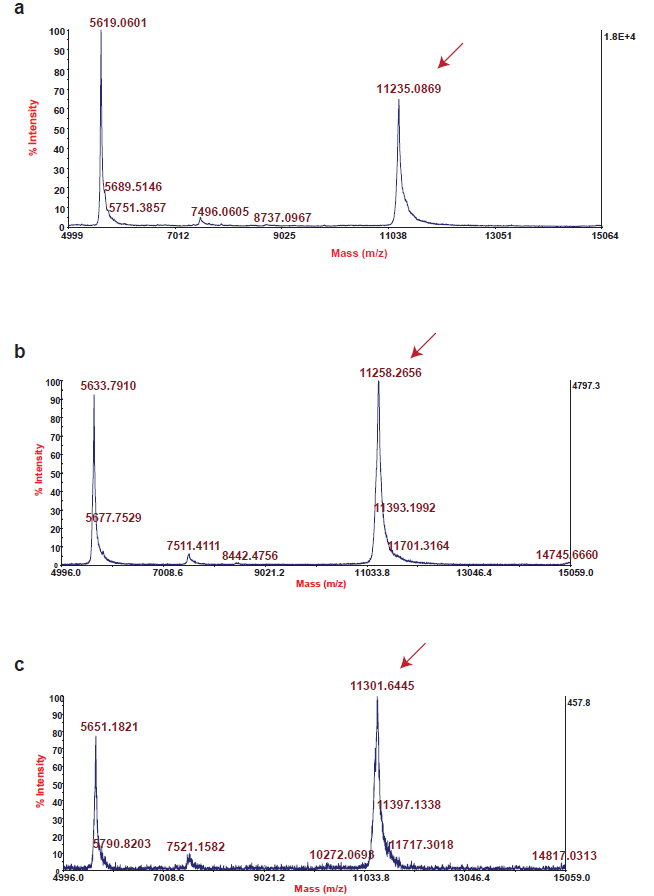
**

**Supplementary Fig. 11.**

Mass spectra of histone H4 containing either K20me0 (a) or K20me1 (b) or K20me3 (c). Red arrows point to the peaks pertaining to the three methylation states.

**Supplementary Figure 12.**

(a) 12-202-601 nucleosome arrays reconstituted with different human histone constructs analyzed by 1.1% agarose gel at the saturation point corresponding to 12 nucleosomes per 12-202 bp DNA array template. (b) AUC-SV titration curves for the 12-202 arrays in the presence of varying amount of Mg^2+^. (c) van Holde-Weischet curves showing the boundary distribution curves for the 12-202 arrays with the different methylated H4K20 histone constructs in the presence of 2.0 mM Mg^2+^. (d) Representative van Holde-Weischet curves. The boundary fraction versus s_20,w_ for 12-202 arrays at varying salt concentrations for the different histone H4K20 methylation constructs in the presence of MgCl_2_ is displayed. For all constructs, two to three independent measurements were performed and the average s_20,w_ obtained from the 20–80% range of the boundary resulted in the values displayed in (b). (e) Precipitation curves of 12-202 arrays with methylation on H4K20. (f) EC_50_ values of the 12-202 arrays. Data are presented as mean values +/- SD. One-way ANOVA analysis (two-sided test) for different arrays was shown. n = 3 independent experiments for all 12-202 arrays. ns, not significant, p ≥ 0.05; *, 0.01 ≤ p <0.05; **, 0.001 ≤ p < 0.01. p = 0.0192 for me0 vs me1, and p = 0.0058 for me1 vs me3. (g) Table showing mean ± SD of EC_50_ values presented in (f). Source data are provided as a Source Data file.

**Supplementary Fig. 13.**

(a) Precipitation curves of 15-197-601 and 16-187-601 arrays with methylation on H4K20. Array samples were diluted with TEN 0.01 buffer until optical density at 260 nm reached 2 and then titrated with equal volume of buffers containing double the final concentration of Mg^2+^. The absorbance of the supernatant was measured at 260 nm after centrifugation. (b) EC_50_ values of the 15-197-601 and 16-187-601 arrays. Data are presented as mean values +/- SD. One-way ANOVA analysis (two-sided test) for different arrays was shown. ns, not significant, p ≥ 0.05; *, 0.01 ≤ p <0.05; **, 0.001 ≤ p < 0.01; ***, 0.0001 ≤ p < 0.001; ****, p < 0.0001. For 15-197 array, n = 4 for K20me0 and K20me3 array; n = 3 for K20me1 array; p<0.0001 for me0 vs me1, p = 0.0009 for me0 vs me3, and p = 0.0009 for me1 vs me3. For 16-187 array, n = 4 K20me0 and K20me1 arrays; n = 3 for K20me3 array; p = 0.0040 for me0 vs me1, p = 0.0344 for me0 vs me3, and p = 0.0002 for me1 vs me3. The efficiency of Mg^2+^ to precipitate the array was characterized by the average Mg^2+^ concentration at 50% precipitation of the array (EC_50_) calculated from at least three measurements. The K20me1 array requires more Mg^2+^ to self-associate and result in aggregation. (c) Table showing mean ± SD of EC_50_ values presented in (b). Source data are provided as source data file.

**Supplementary Fig. 14.**

(a) Overlaid 2D 13C-13C DARR spectra of 15-mer nucleosome arrays containing ^13^C, ^15^N labeled WTH4 (red), H4K20me1 (blue) and H4K20me3 (black). (b & c) Overlaid 2D NCA (b), and NCO (c), correlation spectra of 15-mer nucleosome arrays containing ^13^C, ^15^N labeled WTH4 (red), H4K20me1 (blue) and H4K20me3 (black).

**Supplementary Fig. 15.**

(a) Normalized site-specific NCA peak intensities of all identified H4 residues. (b) Normalized site-specific NCO peak intensities of all identified H4 residues. All peak intensities were normalized to the highest peak intensity in the corresponding dataset. Error bars were derived from the RMSD values of noises of the corresponding spectra.
